# Identification of two *fnr* genes and characterisation of their role in the anaerobic switch in *Sphingopyxis granuli* strain TFA

**DOI:** 10.1038/s41598-020-77927-w

**Published:** 2020-12-03

**Authors:** Yolanda Elisabet González-Flores, Rubén de Dios, Francisca Reyes-Ramírez, Eduardo Santero

**Affiliations:** Departamento de Biología Molecular e Ingeniería Bioquímica, Centro Andaluz de Biología del Desarrollo, CSIC, Universidad Pablo de Olavide, Junta de Andalucía, Carretera de Utrera, Km. 1, 41013 Seville, Spain

**Keywords:** Microbiology, Bacteria, Bacterial physiology, Bacterial transcription

## Abstract

*Sphingopyxis granuli* strain TFA is able to grow on the organic solvent tetralin as the only carbon and energy source. The aerobic catabolic pathway for tetralin, the genes involved and their regulation have been fully characterised. Unlike most of the bacteria belonging to the sphingomonads group, this strain is able to grow in anoxic conditions by respiring nitrate, though not nitrite, as the alternative electron acceptor. In this work, two *fnr-*like genes, *fnrN* and *fixK*, have been identified in strain TFA. Both genes are functional in *E. coli* and *Sphingopyxis granuli* although *fixK*, whose expression is apparently activated by FnrN, seems to be much less effective than *fnrN* in supporting anaerobic growth. Global transcriptomic analysis of a Δ*fnrN* Δ*fixK* double mutant and identification of Fnr boxes have defined a minimal Fnr regulon in this bacterium. However, expression of a substantial number of anaerobically regulated genes was not affected in the double mutant. Additional regulators such *regBA*, whose expression is also activated by Fnr, might also be involved in the anaerobic response. Anaerobically induced stress response genes were not regulated by Fnr but apparently induced by stress conditions inherent to anaerobic growth, probably due to accumulation of nitrite and nitric oxide.

## Introduction

A great number of bacteria show a nutritional versatility that allow them to use multiple molecules as substrates including a wide variety of organic pollutants of natural or anthropogenic origin, many of which are recalcitrant. The reason for this extraordinary flexibility mainly lies in the variety of metabolic genes coded in their genomes but also in the way their regulation networks detect environmental conditions and adjust their cellular physiology to the constant changes in them.

Among these bacteria, members of the sphingomonad group, which comprises several genera of the *Sphingomonadaceae* Family^1,2^, belonging to the Class *α-proteobacteria*, have received particular attention due to their genomic plasticity, ubiquity and metabolic versatility. Plasticity since their genomes are very variable in plasmid content, organization and size^3^ and show many genomic islands and prophages that suggest frequent events of incorporation of external DNA into their genomes. Ubiquity is reflected in that they are found in most of the environments, although they play a particularly important role in nutrient cycling in marine environments^4^ due to its excellent adaptation to oligotrophic environments^5^. Their metabolic versatility includes the ability of many of them to degrade different recalcitrant molecules such as polycyclic aromatic hydrocarbons^6^ or xenobiotic compounds such as lindane^7^, frequently harbouring this biodegradative capacity in plasmids^8^.

*Sphingopyxis granuli* strain TFA is one representative example of a bacterium able to degrade a recalcitrant molecule, in this case, tetralin (1,2,3,4-tetrahydonaphthalene), a bicyclic molecule with an aromatic and an alicyclic ring sharing two carbon atoms. Only a few strains have been reported to metabolise tetralin and its biodegradation pathway have been characterised in just two of them^9–11^. TFA is the only strain in which the tetralin biodegradation pathway and its sophisticated regulatory network has been fully characterised at a biochemical and genetic level^11–13^ (and references therein).

The genome sequence of *Sphingopyxis granuli* strain TFA revealed features typical of oligotrophic bacteria, biodegradation genes in addition to the ones involved in tetralin biodegradation-which might confer additional uncharacterised biodegrading capabilities to this strain. Besides its capability of using tetralin, TFA exhibits another interesting feature, which is its ability to grow anaerobically by respiring nitrate. As suspected by its genome sequence it was shown that only nitrate was used as a terminal electron acceptor during anaerobic respiration and that nitrate reduction leads to a continuous accumulation of nitrite^14^. This last feature that allows this bacterium to colonise anoxic environments, is particularly relevant since *Sphingopyxis* was considered a strictly aerobic genus^1^, and no anaerobic growth of other *Sphingopyxis* strains has been demostrated so far, although nitrate reduction to nitrite has also been reported in two other *Sphingopyxis* strains^15,16^.

The global transcriptomic response of TFA to anaerobiosis has recently been reported^17^, and showed that in addition to genes conventionally regulated by anaerobiosis, such as nitrate respiratory genes, many other genes potentially involved in response to different stress conditions, including genes responding to nitrosative stress, were induced. These results led us to suggest that the anoxic condition is a hostile environment for TFA and that nitric oxide might be accumulating to sufficient concentration to cause damage in the growing bacteria.

The transcription factor Fnr (fumarate and nitrate reduction regulator) is the major global regulator that controls activation of gene expression when cells become anaerobic^18^. Fnr most frequently activates transcription by binding to conserved binding sites centred at different positions upstream of Class I or Class II promoters^19,20^, although it may also repress transcription. Fnr-like proteins have been found and their function characterised in many different bacteria, including members of *α-Proteobacteria*, although in this Class of bacteria other regulators such FixLJ, FixK, RegBA or CrtJ have also been reported to control responses to anaerobiosis^21,22^.

The genome of strain TFA harbours two *fnr-*like genes, annotated as *fnrN* and *fixK*, and *regBA* genes coding for a regulatory two-component system. In this work we show that both *fnr-*like genes are functional in both *Sphingopyxis granuli* and *E. coli*, although *fixK* is less efficient in activating transcription and is not required for anaerobic growth in *Sphingopyxis granuli.* Transcriptomic analysis of a Δ*fnrN* Δ*fixK* double mutant and identification of putative Fnr binding sites have led to the definition of a minimal Fnr regulon in this bacterium.

## Results

### Identification of two *fnr*-like genes in the TFA genome, *fnrN* and *fixK*

Two putative regulatory genes, SGRAN_2447 and SGRAN_3861, annotated as *fnrN* and *fixK*, respectively, were identified in the TFA genome, which coded for products that had evident similarity to Fnr proteins of different bacteria. Alignment of TFA FnrN and FixK proteins and comparison to recognised Fnr regulators of anaerobiosis from other species (Suppl. Fig. S1) representative of *α-*, *β-* and *γ-Proteobacteria* revealed that both proteins had the four conserved cysteine residues in the ligand binding domain that actually bind the iron-sulphur ligand.

The third cysteine in both proteins is displaced two residues in relation to the archetypal Fnr proteins from *β-* and *γ-Proteobacteria*. This slightly modified arrangement is characteristic of the FnrN subgroup of the CRP-FNR superfamily of transcriptional regulators that seems to be exclusively represented in *α-Proteobacteria* (Suppl. Fig. S1 and^21,23^). FnrN and FixK, which also belong to the CRP-FNR superfamily, are key regulators in the expression of genes involved in nitrogen fixation, photosynthesis, and degradation of aromatics, hydrogen uptake, and haem biosynthesis. The main difference between them is that FixK members, lack of the conserved N-terminal cysteines required for ligating the sensory iron-sulphur cluster of Fnr proteins, in fact the presence or not of such cysteines is considered to distinguish between FnrN-like and FixK-like regulators. The multiple sequence alignment of Suppl. Fig. S1 also include a FixK protein from *Bradyrhizobium diazoefficiens* (formerly *japonicum*), which similarly to FixK from TFA, encodes an Fnr-type protein belonging to the FnrN group in spite of its name. The AadR regulator from *Rhodopseudomonas palustris* previously known as a FnrN family member with its altered spacing between the cysteines it is also included^21,23^. This suggests that both FnrN and FixK from TFA can bind an iron-sulphur cluster. Apart from the cysteines, the highest identity observed among the different Fnr proteins including FnrN and FixK from TFA, corresponds to the helix-turn-helix motif of their C- terminal domain, particularly the highly conserved glutamic acid, serine and arginine residues in the second helix that are important for the recognition of the Fnr box, indicative that they have a similar DNA recognition sequence (Suppl. Fig. S1).

### Heterologous complementation of an *E. coli fnr* mutant with *fnrN* and *fixK*

**Figure 1:**
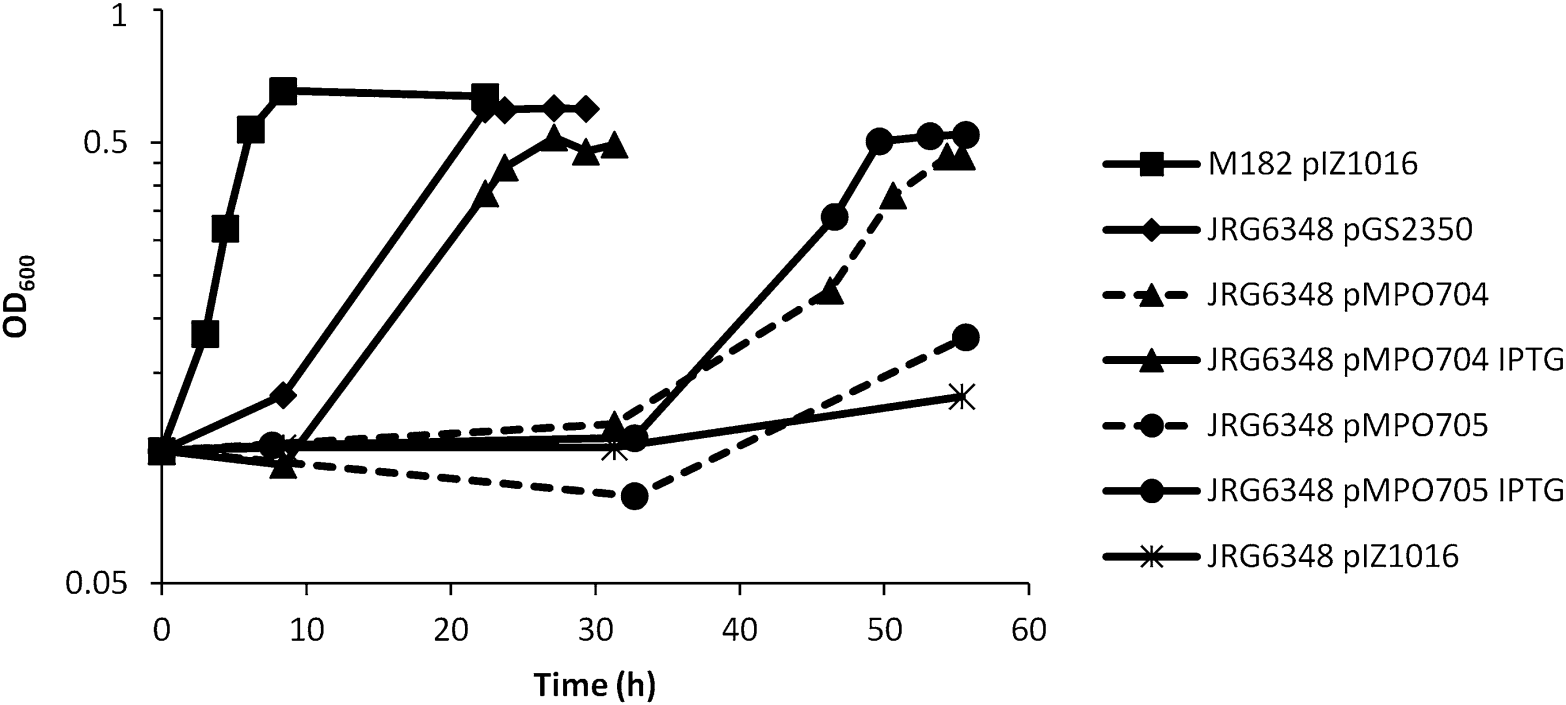
Functional complementation of Δ*fnr Escherichia coli* with TFA genes *fnrN* and *fixK*. Absorbance at 600 nm (OD_600_) is shown over time for *E. coli* WT strain M182 with the empty plasmid (pIZ1016; squares), the Δ*fnr E. coli* mutant JRG6348 bearing the empty plasmid (pIZ1016; star) and the same strain complemented with plasmids carrying either *E. coli fnr* gene (pGS2350; diamonds), *S. granuli fnrN* gene (pMPO704; triangles), or *S. granuli fixK* gene (pMPO705; circles). The complementations with pMPO704 and pMPO705 were performed in the presence (continuous line) or absence (dotted line) of the inducer IPTG.

The two genes identified in the TFA genome putatively coding for Fnr-like proteins were amplified by PCR from genomic DNA and cloned in the broad host range expression vector pIZ1016 in such a way that the genes could be transcribed from the heterologous *tac* promoter present in the vector and their expression induced by IPTG (isopropyl β-d-1-thiogalactopyranoside). The resulting plasmids were transferred to the *fnr* deletion mutant of *E. coli* JRG6348, and transformants bearing each plasmid were tested for growth under conditions for anaerobic respiration, using glycerol as a non-fermentable carbon source and nitrate as the terminal electron acceptor. As shown in Fig. 1, the wild type strain M182 bearing the empty vector pIZ1016 was able to grow and reached stationary phase in less than 9 h. However, the *fnr* isogenic mutant was unable to grow at all after 55 h. This mutant was effectively complemented for growth with the plasmid pGS2350 bearing the *E. coli fnr* gene used as a positive control. When this mutant bore the *fnrN* gene from the strain TFA, a slow growth was observed after 30 h of incubation. The growth clearly improved in the presence of the IPTG inducer, when transcription of *fnrN* from the *tac* promoter increased. In the case of complementation with *fixK*, very little growth was observed in the absence of IPTG, although very obvious growth was observed in the presence of IPTG. These results clearly indicate that both *fnrN* and *fixK* from strain TFA are functional and can achieve inter-Class complementation of the *E. coli fnr* mutant growth deficiency under anaerobic respiration conditions. However, it appears that *fnrN* is more efficient than *fixK* at least in *E. coli*.

### Phenotype analysis of single and double *fnrN* and *fixK* mutants

**Figure 2:**
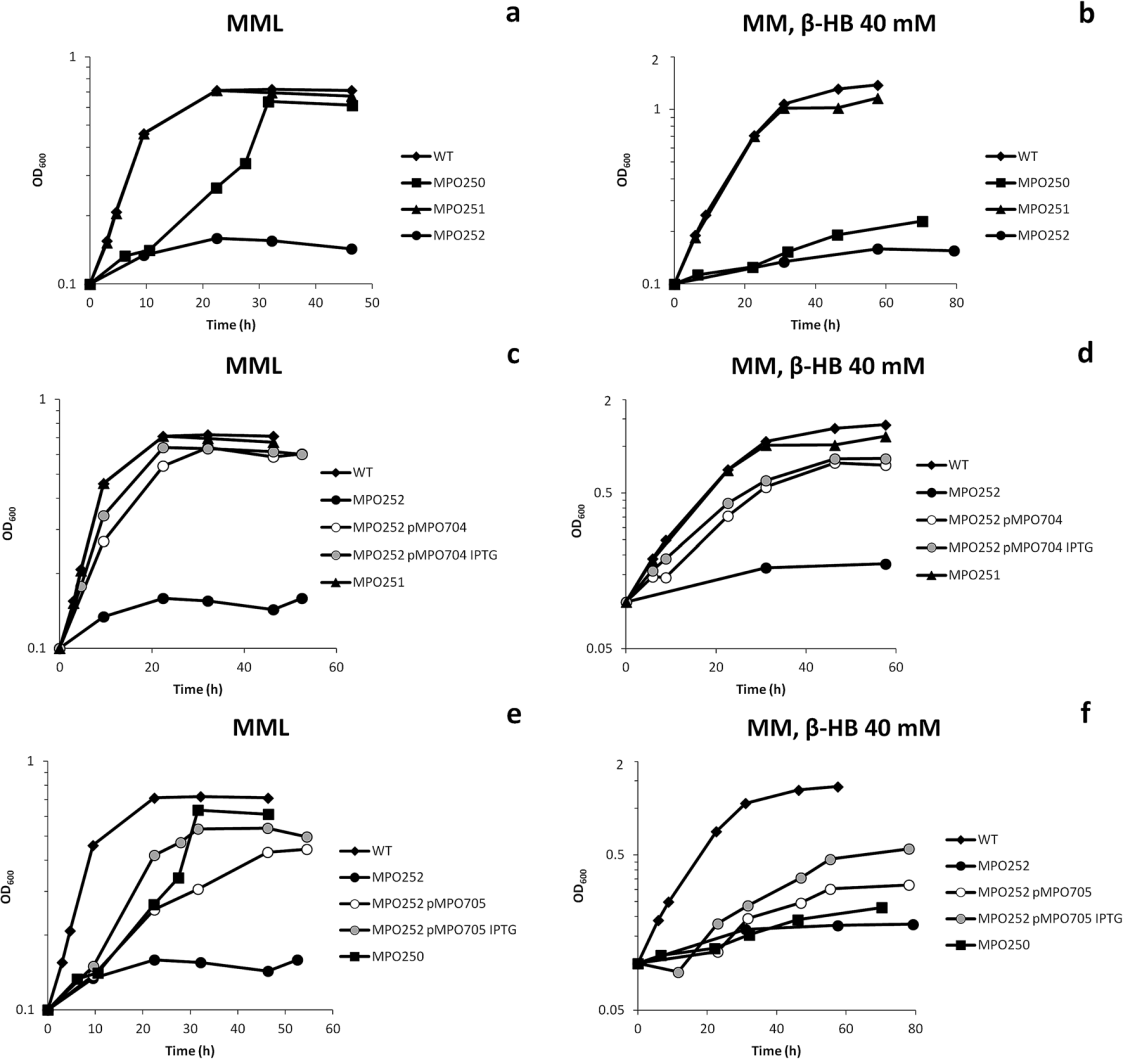
Anaerobic growth of Δ*fnrN* and Δ*fixK* mutants of TFA in different media. Growth was tested both in rich (**a**, **c** and **e**) or mineral medium (**b**, **d** and **f**). Absorbance at 600 nm (OD_600_) is shown over time for the WT strain (black diamonds), the single Δ*fnrN* (MPO250; black squares), and Δ*fixK* mutants (MPO251; black triangles), and for the double Δ*fnrN*Δ*fixK* mutant (MPO252; black circles) in (**a**) and (**b**), for the double mutant MPO252 complemented with the plasmid carrying *fnrN* (pMPO704) in the absence (white circles) and presence (grey circles) of the inducer IPTG in (**c**) and (**d**), and for the double mutant MPO252 complemented with the plasmid carrying *fixK* (pMPO705) in the absence (white circles) and presence (grey circles) of the inducer IPTG in (**e**) and (**f**).

To establish the role of each gene in anaerobic growth of the strain TFA, single deletion mutants in each gene and a double Δ*fnrN* Δ*fixK* deletion mutant were constructed by double recombination events, as previously described^17^ (see “Methods”) and their anaerobic growth tested in rich MML medium (mineral medium plus 2 g of tryptone liter^−1^ and 1 g of yeast extract liter^−1^) and minimal medium with β-HB (β-hydroxybutyrate) as the only carbon source. The Δ*fixK* mutant MPO251 showed very similar growth pattern to the wild type strain in both media, which indicated that FixK is dispensable for anaerobic growth (Fig. 2a,b). On the other hand, the Δ*fnrN* mutant MPO250 showed an evident growth deficiency in anaerobiosis. In rich MML medium, it was finally able to grow and to reach a similar cell density, although growth was slower. The growth deficiency was even more evident in minimal medium, where it was barely able to duplicate after 60 h (Fig. 2a,b). Finally, the double Δ*fnrN* Δ*fixK* mutant MPO252 was unable to grow in either medium (Fig. 2a,b). As expected when nitrate and nitrite concentrations were measured in mineral medium with β-HB as carbon and energy source, the *ΔfnrN* and *ΔfnrNΔfixK* mutant strains did not excrete nitrite and nitrate concentration remained constant. In contrast, in the Δ*fixK* mutant a progressive reduction of nitrate concentration and an almost stoichiometric accumulation of nitrite was obtained, similarly to what has been previously observed in the wild type strain (Suppl. Fig. S2).

The double Δ*fnrN* Δ*fixK* mutant was transformed with the plasmids bearing *fnrN* or *fixK* transcribed from the *tac* promoter, and anaerobic growth of the transformants was tested in both media in the absence or presence of IPTG. As shown in Fig. 2c,d, *fnrN* very efficiently complemented the growth phenotype of the double mutant in both media and IPTG had very little effect, thus suggesting that basal transcription from the strong *tac* promoter was sufficient for complementation. On the other hand, *fixK* seems to compensate the lack of FnrN function better in rich that in minimal medium, since complementation with *fixK* partially complemented growth in MML medium, requiring addition of IPTG to improve complementation (Fig. 2e), whereas it barely complemented growth in minimal medium (Fig. 2f).

Taken together, these results indicate that both *fnrN* and *fixK* are functional *fnr*-like genes that play a role in anaerobic growth of *S. granuli* strain TFA. However, though functional, it appears that *fixK* just plays a secondary role in anaerobic growth of this bacterium.

### Expression kinetics of *fnrN* and *fixK* genes after transfer to anaerobic conditions

**Figure 3:**
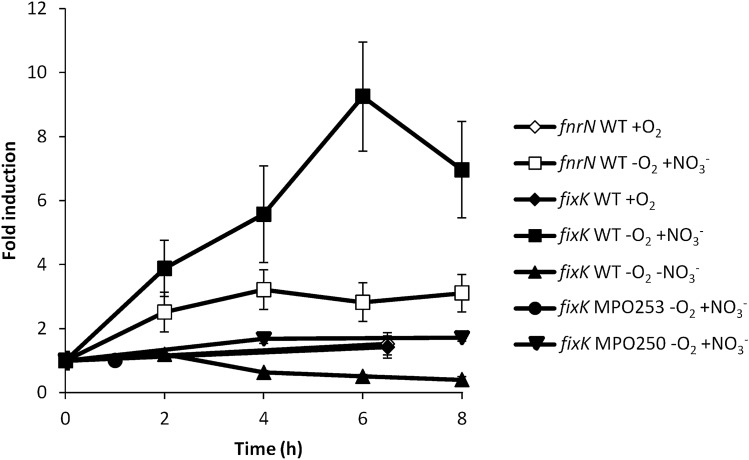
Induction kinetics of *fnrN* and *fixK* genes. Induction kinetics of *fnrN* (white) and *fixK* (black) are represented. Aerobic conditions in the WT strain are shown by diamonds, anaerobic conditions with 20 mM nitrate in the WT strain by squares, anaerobic conditions without nitrate in the WT strain by triangles, anaerobic conditions with 20 mM nitrate in the Δ*narG* mutant MPO253 by circles and anaerobic conditions with 20 mM nitrate in the Δ*fnrN* mutant MPO250 by inverted triangles. Fold change induction of each gene over time with respect to time 0 is shown and graphic represents the mean ± SD of 3–4 technical replicates.

Previous differential RNA-sequencing (dRNA-seq) analysis under anaerobic conditions^17^ suggested that both *fnrN* and *fixK* could be upregulated under anaerobic conditions. In order to confirm induction of these genes upon transference to anaerobic conditions, induction kinetics were performed by real-time quantitative polymerase chain reaction (RT-qPCR) after anaerobic incubation in minimal medium. As shown in Fig. 3, expression of *fnrN* was weakly upregulated up to threefold within the first 4 h after the switch to anaerobic conditions. Induction of *fixK* was higher, reaching up to tenfold upregulation within the first 6 h. Upregulation of *fixK* was also tested in the Δ*fnrN* mutant, and results suggests that its anaerobic induction was dependent on FnrN. However, since *fixK* was not induced in the *narG* mutant either (*narG* is part of the *narUGHJI* operon encoding for a membrane-bound nitrate reductase complex), nor in a WT strain in the absence of nitrate, conditions in which these strains cannot grow anaerobically, it seems that *fixK* activation by FnrN is in somehow dependent on anaerobic growth. According to that *fixK* is assigned to the gene category 2 (see below).

### Fnr-dependent expression of anaerobically regulated genes

In order to determine which genes could be regulated directly by the Fnr proteins of TFA and define its Fnr regulon, a dRNA-seq analysis in the double Δ*fnr* mutant MPO252 was performed. As this mutant is unable to grow anaerobically, besides the genes regulated by Fnr proteins, the expression of a number of genes would be affected as a consequence of the lack of respiration and growth. For this reason, we also performed a dRNA-seq analysis in the Δ*narG* mutant MPO253 unable to respire and grow anaerobically but not affected in regulation. By comparing these two mutants, we intended to discern which genes were regulated directly or indirectly by Fnr protein and which ones as a consequence of lack of respiration and anaerobic growth. The results of these dRNA-seq analyses are shown in the Supplementary Table S1.

In order to be able to meaningfully compare expression in the WT and the mutants, we first analysed the genes that were not affected in anaerobic conditions or affected less than twofold with respect to aerobic conditions (Suppl. Table S1, first tab). We observed that expression of most of these genes not regulated by anaerobiosis was neither affected in the Δ*fnr* nor in the Δ*narG* mutants. Altered expression in the few affected genes could be a consequence of changes in the physiology of the mutant cells as well as differences in the conditions of the dRNA-seqs. Therefore, these previous verification validated the comparisons between the different dRNA-seqs.

Expression in the WT strain was then compared to that in the mutants, by analysing which of the genes affected in anaerobic conditions in the WT showed a large difference in fold-change in the mutants, in comparison with the rest of genes of the genome. Out of the 478 genes that showed differential expression in anaerobiosis of at least threefold, 102 of them had an expression affected in the mutants less than twofold. However, a large number of them actually showed a substantial change in their expression levels (Suppl. Table S1, second tab). They were grouped in different categories according to their regulation pattern.

#### Category 1: Anaerobically induced genes that are more expressed in Δ*narG* than in Δ*fnr* mutant

A total of 26 genes, which were induced in anaerobic conditions in the WT strain at least threefold, had lost this induction in anaerobic conditions in the double Δ*fnr* mutant MPO252 but did not lose it or lost it to a lesser extent in the Δ*narG* mutant MPO253 (at least threefold more expressed in Δ*narG* than in Δ*fnr*), being these the genes which could be most probably regulated by anaerobiosis and Fnr proteins in TFA (Suppl. Table S1, third tab).

**Figure 4:**
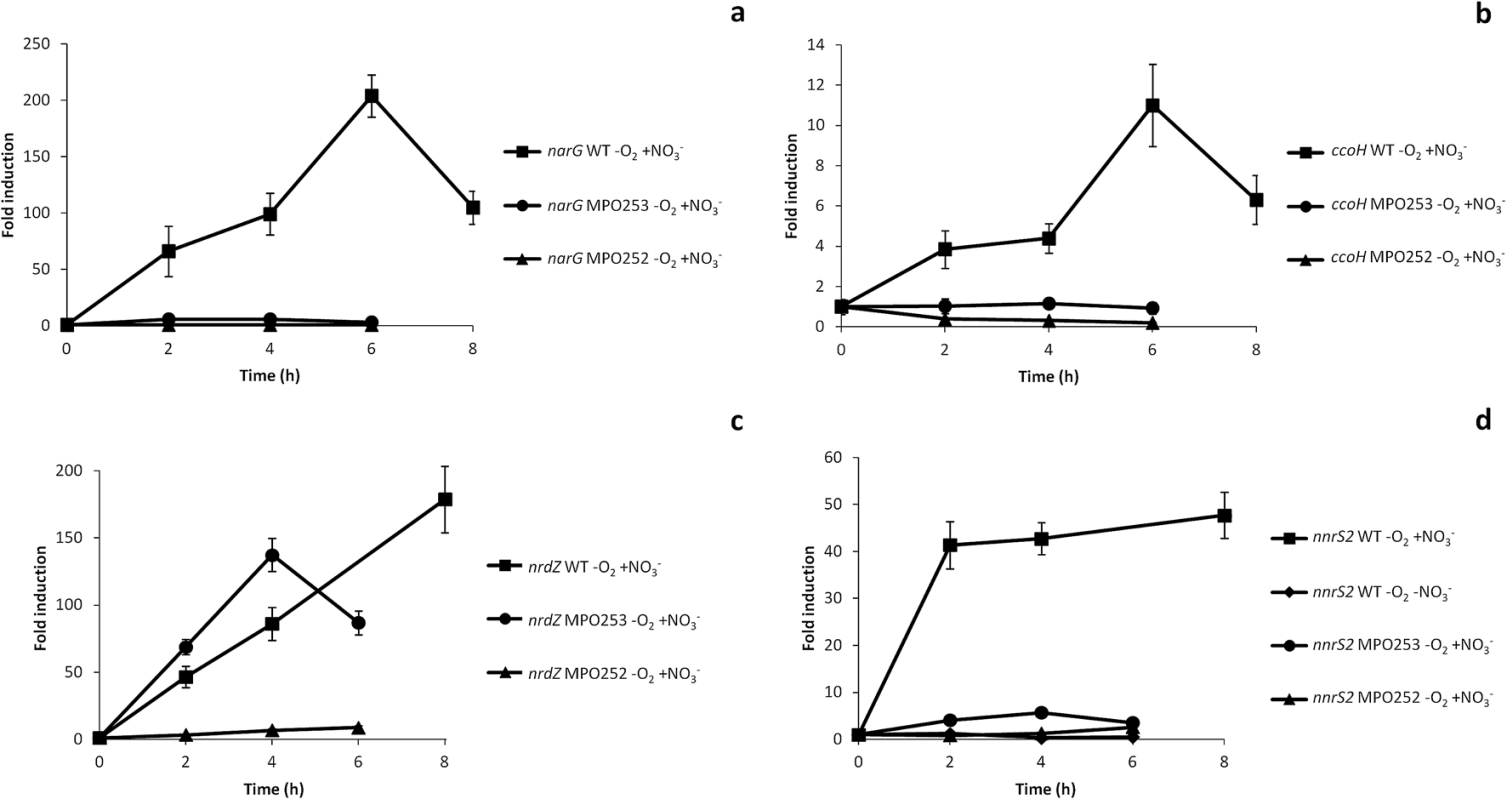
Induction kinetics of genes belonging to category 1. Figure shows the induction kinetics of *narG* (**a**), *ccoH* (**b**), *nrdZ* (**c**) and *nnrS2* (**d**) under anaerobic conditions in the WT strain with nitrate (squares), in the WT without nitrate (diamonds), in the Δ*narG* mutant MPO253 with nitrate (circles) and in the Δ*fnrN*Δ*fixK* mutant MPO252 with nitrate triangles. Fold change induction of each gene over time with respect to time 0 is shown and graphic represents the mean ± SD of 3–4 technical replicates.

As expected, most *nar*, *moa* or *moe* genes that are part of the large operon *narUGHJInifMmoaADEBCmoeA* (SGRAN_3846–3856), coding for the respiratory nitrate reductase and its molybdenum cofactor, belong to this group. Nevertheless, their expression in the Δ*narG* mutant was substantially lower than in the WT. Induction kinetics of *narG* (Fig. 4a), showed that this gene was very slightly induced in the Δ*narG* mutant (a maximum of sixfold), as compared to the 200-fold maximum induction in the WT strain, which suggested that full induction of the *nar* operon also needs nitrate respiration and growth. This pattern was also observed in the final part of the *cco* operon (*ccoHIS*; SGRAN_2454-2456), coding for an alternative *cbb3*-type terminal oxidase, consistent with the fact that this type of oxidases have been reported to have high affinity for oxygen, thus allowing respiration when oxygen concentrations are very low^24,25^. Induction kinetics of *ccoH* gene by RT-qPCR (Fig. 4b) showed that expression of this gene was actually repressed up to fivefold in the Δ*fnr* mutant, while in Δ*narG* its expression was constant along the anaerobic incubation time. Again, it appears that high induction of these genes need nitrate respiration. The proximal part of this operon, *ccoNOQPG* (SGRAN_ 2449–2452) followed a similar pattern, less expressed in Δ*fnr* than in WT or Δ*narG* (Suppl. Table S1, first tab), but its anaerobic induction was very low due to their high level of expression in aerobiosis^17^.

The ribonucleoside reductase *nrdZ* (SGRAN_3862), an O_2_-independent ribonucleotide reductase that help cells adapt to anaerobic conditions^26^, and is essential for DNA synthesis in anaerobiosis^27^, was also included in this group, since it was 19-fold more expressed in Δ*narG* than in Δ*fnr*. The induction kinetics showed that this gene was substantially induced in Δ*narG*, but there was no induction in the Δ*fnr* mutant (Fig. 4c). These results confirm that *nrdZ* could be actually regulated by the Fnr proteins of TFA and does not need nitrate respiration. However, it appears that the presence of nitrate is necessary for induction, since it was previously reported that WT TFA strain could not anaerobically induce this gene in the absence of nitrate^17^.

Interestingly, SGRAN_3353, coding for a Universal Stress Protein similar to UspA, reported to protect cells against stress situations as well as DNA damage^28^, was also in this category although in this case, expression in the Δ*narG* mutant was huge, much higher than in the WT. A similar pattern was also observed for *ilvX* (SGRAN_2714), encoding a putative alternative acetolactate synthase subunit^29^, *cybB* (SGRAN_0602), encoding the cytochrome *b561*^30^ or *hemN2* (SGRAN_2458), encoding an oxygen independent Coproporphyrinogen-III oxidase involved in anaerobic biosynthesis of heme^31^. However, these genes were apparently poorly induced by anaerobiosis in the WT strain (≤ twofold). These genes might be induced by starvation stress, but they apparently also need Fnr proteins for induction.

A gene of this category, the *nnrS2* regulatory gene reported to protect cells under NO stress in *V. cholerae* and *S. oneidensis*^32^, is part of a Cupin-*norBnnrS2* operon (SGRAN_3802–3800) that was also induced in aerobiosis by NO to the same extent or even more than by anaerobiosis^17^. Induction kinetics (Fig. 4d) show that this gene was induced very fast in anaerobic conditions with nitrate in the WT strain, almost reaching its maximum induction (40-fold) in the first two hours of growth, while in the absence of nitrate it was not induced but even repressed down to threefold. In Δ*narG* mutant *nnsR2* gene was slightly induced up to sixfold whilst in the Δ*fnr* mutant it was not induced at all. This suggests that Fnr might activate its expression but full induction of this gene requires nitrate being respired, possibly to produce NO with the nitrate reductase activity^17,33^.

Finally SGRAN_4065-4066 and 4071), encoding for different dehydrogenases also met the requirements to be ascribed to this category.

#### Category 2: Anaerobically induced genes that are downregulated in both Δ*narG* and Δ*fnr* mutants

This is the most numerous category. The dRNA-seq revealed that 107 genes that were induced in anaerobic conditions in the WT strain had lost this induction in both the Δ*fnr* double mutant and the Δ*narG* mutant (Suppl. Table S1, fourth tab). This category includes the *fnr-*like *fixK* (SGRAN_3861). This ascription cannot be done on the basis of the dRNA-Seq results because of the Δ*fixK* deletion in the double Δ*fnr* mutant used in this analysis but Fig. 3 indicates that it is not expressed in the Δ*fnrN* or the Δ*narG* mutants.

Many genes of this category are potentially involved in stress protection or detoxification such as different operons involved in metabolite transport across the membrane, Fe-S clusters biogenesis or repair, production of the ectoin osmoprotectant, phasin associated to polihydroxyalcanoate granules, error-prone DNA repair, or *lsfA* that codes for a peroxidase^34^. Previous induction kinetics in anaerobiosis of some of these genes showed that their induction in anaerobic conditions was slow^17^. These data together suggest that induction of these genes is responding to conditions created while growing under anaerobiosis, rather than to a real induction by anaerobiosis.

Inside this group, there are genes that were also aerobically induced by NO^17^.

**Figure 5:**
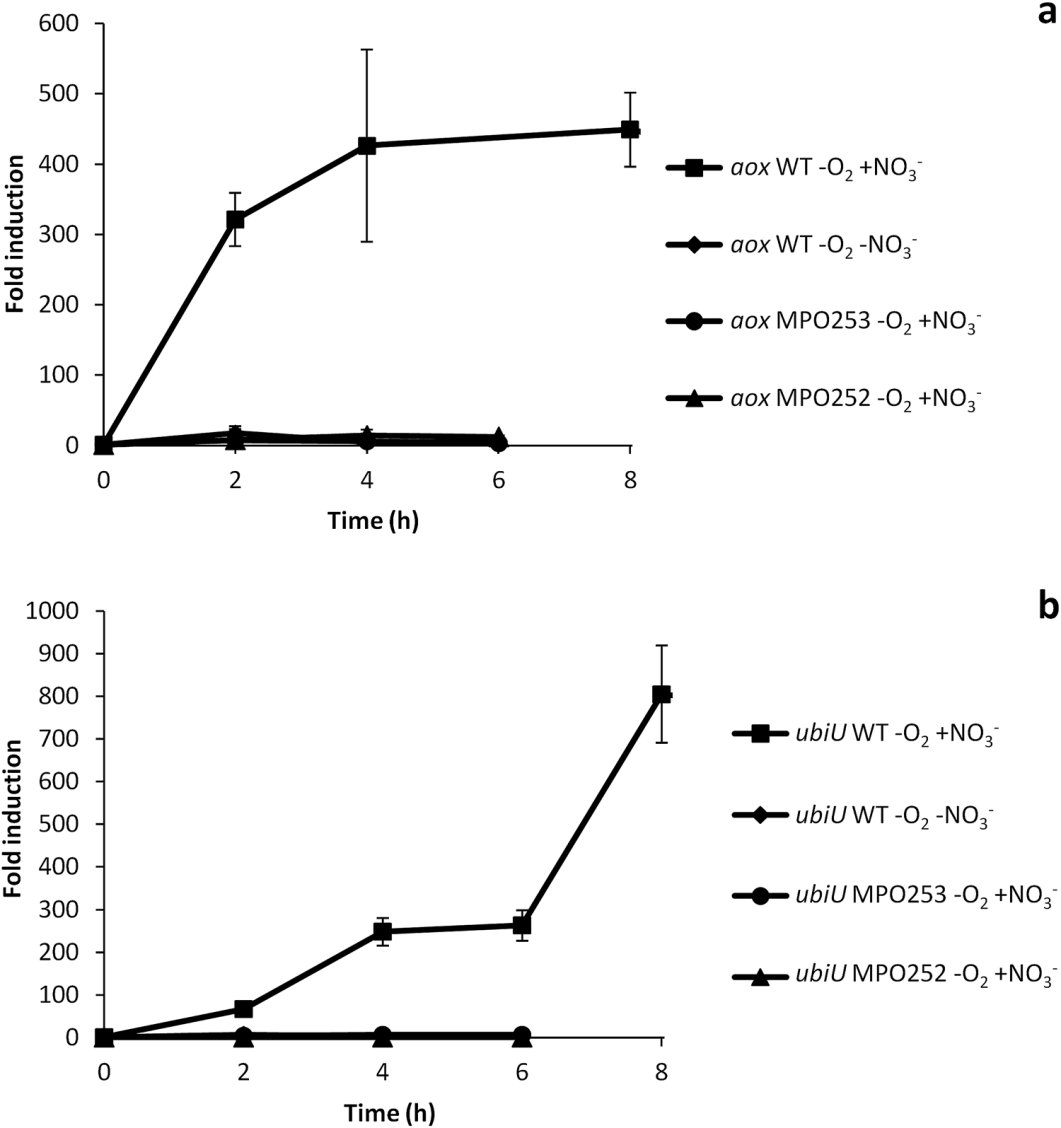
Induction kinetics of genes belonging to category 2. Figure shows the induction kinetics of *aox* (**a**) and *ubiU* (**b**) under anaerobic conditions in the WT strain with nitrate (squares), in the WT without nitrate (diamonds), in the Δ*narG* mutant MPO253 with nitrate (circles) and in the Δ*fnrN*Δ*fixK* mutant MPO252 with nitrate (triangles). Fold change induction of each gene over time with respect to time 0 is shown and graphic represents the mean ± SD of 3–4 technical replicates.

Within this category there is a sub-group of genes whose expression in the Δ*narG* mutant was even lower than that in the Δ*fnr* mutant. Some representatives examples are the SGRAN_1370-1368 operon (Fe-S cluster biogenesis), *ytfE*, coding for the Fe-S cluster repair protein, SGRAN_3394 coding for the preprotein translocase subunit TatC and *aox*, coding for an NO-insensitive alternative oxidase. Previous induction kinetics of *ytfE* showed that it was induced slowly in anaerobic conditions in the WT strain^17^. However, *aox* induction kinetics was really fast and complete after 4 h in anaerobiosis, as shown in Fig. 5a, thus suggesting a different regulation kinetics from *ytfE*. A possible explanation is that in Δ*fnr* these genes could be induced to some extent by NO produced by the basal activity of the nitrate reductase, which is completely lost in Δ*narG* as one of the subunits of this enzyme is mutated. Therefore, this subgroup of genes would probably be induced by NO (i.e. *aox*) or by the nitrosative damage caused by its accumulation (i.e. *ytfE*).

Other genes of this category include *moeB* (SGRAN_0222), an unlinked gene involved in Mo cofactor biosynthesis, most of the components of the *cyoABCDEregBA* operon, (SGRAN_2997-_2991) encoding a *bo3*-type quinol oxidase electron transfer chain and the redox responsive *regBA* two-component regulatory system^18^. *yhbTUV* operon (SGRAN_3858-_3860) is one of the most induced operons in anaerobiosis^17^ and it is involved in the recently described O_2_-independent ubiquinone biosynthesis pathway, proposed to be renamed *ubiTUV*^35^, and it is essential for denitrification in *Pseudomonas*^36^. Althought dRNA-Seq showed that expression of the distal genes of this operon was still maintained to some extent in the Δ*narG* mutant, the , induction kinetics of *ubiU* in Δ*fnr* and in Δ*narG* mutants (Fig. 5b) showed that it was not induced in any of the mutants, therefore ascribed to this category.

#### Category 3: Anaerobically induced genes that are upregulated in both Δ*narG* and Δ*fnr* mutants

A total of 25 genes, despite being induced in anaerobic conditions in the WT^17^, were even more induced in the Δ*narG* and the Δ*fnr* double mutant (Suppl. Table S1, fifth tab). Genes with this expression pattern included those belonging to the two divergent operons, SGRAN_0596-0593 and SGRAN_0597-0601 involved in sulfur detoxification, which were induced between 5 and tenfold more in the mutants. Others are also related to stress responses such as *trxC* (SGRAN_0625) coding for the antioxidant thioredoxin^37^ or *ahpC* (SGRAN_0624) and *ahpC2* (SGRAN_0626) coding for peroxiredoxins. Although these genes are contiguous, they appear to form independent transcriptional units.

**Figure 6:**
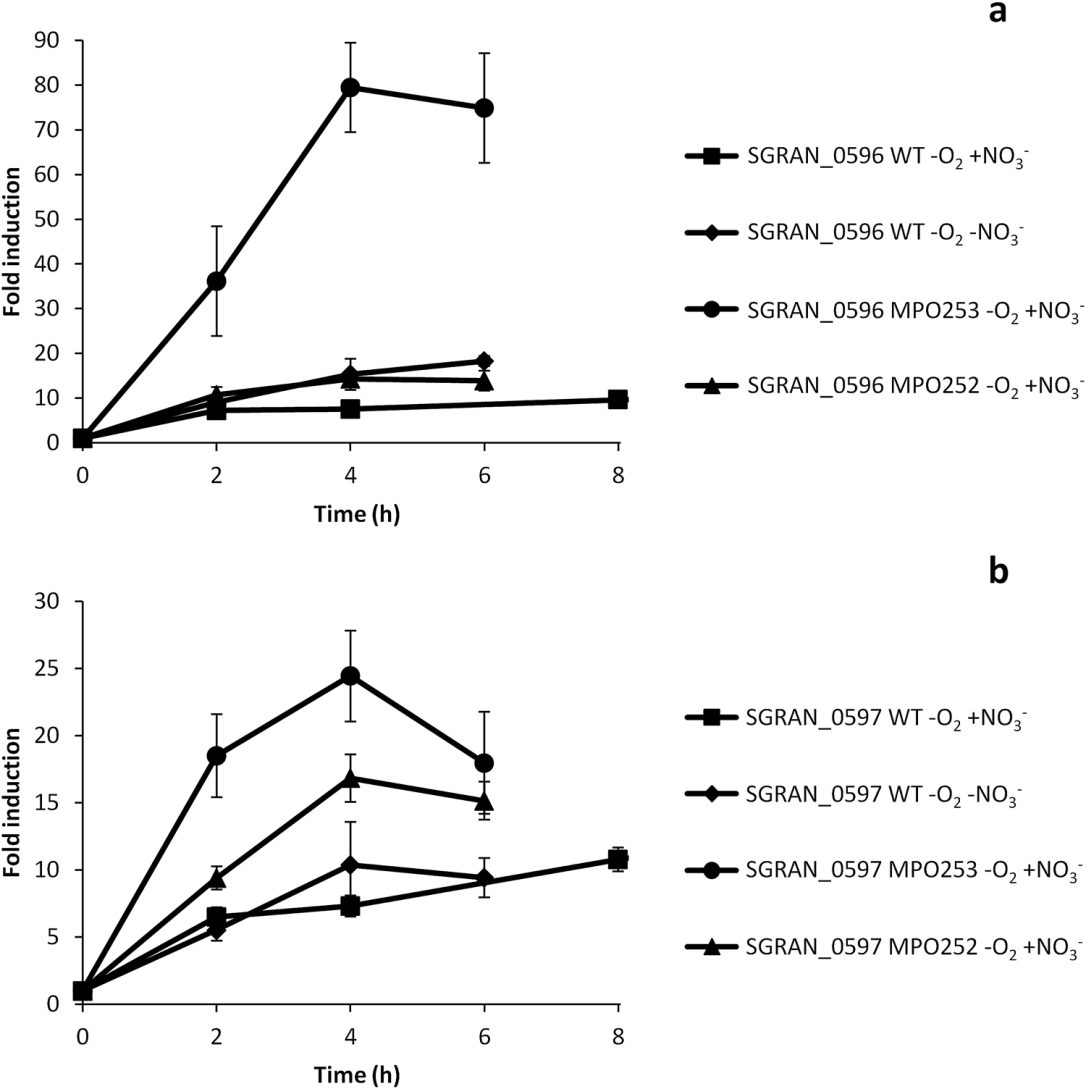
Induction kinetics of genes belonging to category 3. Figure shows the induction kinetics of SGRAN_0596 (**a**) and SGRAN_0597 (**b**) under anaerobic conditions in the WT strain with nitrate (squares), in the WT strain without nitrate (diamonds), in the Δ*narG* mutant MPO253 with nitrate (circles) and in the Δ*fnrN*Δ*fixK* mutant MPO252 with nitrate (triangles). Fold change induction of each gene over time with respect to time 0 is shown and graphic represents the mean ± SD of 3–4 technical replicates.

Induction kinetics by RT-qPCR shown in Fig. 6a, indicate that SGRAN_0596 was induced in anaerobic conditions with nitrate in the WT strain about eightfold within the first 8 h. Intriguingly, induction was higher in the WT without nitrate and in anaerobic conditions with nitrate in the Δ*fnr* mutant, reaching its maximal expression level, 80-fold induction in 4 h, in anaerobic conditions with nitrate in the Δ*narG* mutant. The divergent gene SGRAN_0597 showed very similar induction kinetics to that of SGRAN_0596 in anaerobic conditions with nitrate in the WT (Fig. 6b). This confirms the dRNA-Seq data and indicates that these genes are not regulated at all by Fnr proteins but are somehow induced by a limitation or stress under anaerobic condition, which become greatest when the bacteria could not respire nitrate.

It seems that these genes are induced in conditions where they cannot grow because of lack of energy. However none of them were aerobically induced by carbon starvation or DETA-NO, which should inhibit aerobic respiration^17^.

Interestingly, the *cydA* gene (SGRAN_0616), encoding one subunit of an ubiquinol oxidase, is also ascribed to this category, in agreement with a previous observation indicating that induction of this gene in the WT strain was even higher in the absence of nitrate^17^. Intriguingly, the downstream gene *cydB* encoding the partner subunit of the ubiquinol oxidase is expressed to lower levels in the mutants (Suppl. Table S1, first tab).

#### Category 4: Genes repressed in anaerobiosis that lost this repression in Δ*fnr*

The dRNA-seq analysis revealed that 57 genes previously found to be repressed in anaerobiosis in the WT strain, lost this repression in the Δ*fnr* double mutant (Suppl. Table S1, sixth tab), which suggests that these genes could be repressed by Fnr. Among these genes were the putative flagellar regulator FleQ (SGRAN_4107), most of the flagellar genes and the prepilin peptidase CpaA. Consistently with this, the cell cycle regulator *ctrA*, reported as the master regulator of flagellum biosynthesis genes in *Sphingomonas melonis*^38^, was also upregulated in the mutants. However, practically all of them also lost the anaerobic repression in the Δ*narG* mutant to a similar extent. These results suggest that these genes are not actually repressed by Fnr, being the loss of their repression in the mutants a consequence of their inability to grow.

#### Category 5: Genes more expressed in Δ*fnr* than in WT and Δ*narG*

36 genes were found that were expressed similarly in the WT strain and in Δ*narG* mutant, but induced in Δ*fnr* mutant (Suppl. Table S1, seventh tab). These could be genes actually repressed by Fnr in anaerobiosis. However, none of them showed repression in anaerobic conditions in the WT strain, being some of them even induced in these conditions^17^. Potential function of these genes is heterogeneous and, in most cases, their upregulation in the Δ*fnr* mutant was modest.

**Figure 7:**
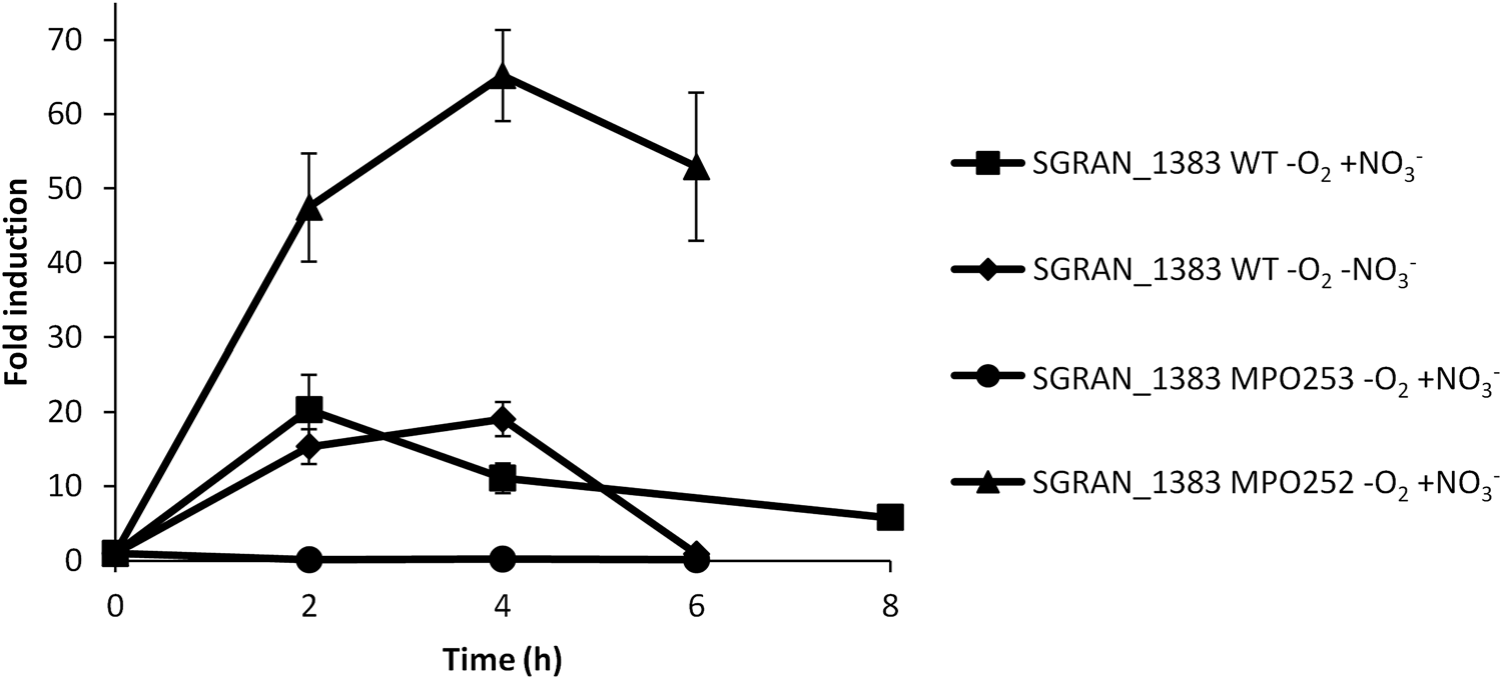
Induction kinetics of SGRAN_1383, belonging to category 5. Figure shows the induction kinetics of this gene under anaerobic conditions in the WT strain with nitrate (squares), in the WT strain without nitrate (diamonds), in the Δ*narG* mutant MPO253 with nitrate (circles) and in the Δ*fnrN*Δ*fixK* mutant MPO252 with nitrate (triangles). Fold change induction of the gene over time with respect to time 0 is shown and graphic represents the mean ± SD of 3–4 technical replicates.

Expression of the most upregulated gene in this category (50-fold in the Δ*fnr* mutant), SGRAN_1383, potentially coding for a membrane protein, was followed by RT-qPCR. As shown in Fig. 7, the gene was induced by anaerobiosis in WT very fast with a maximum expression within 2–4 h, regardless the bacteria could respire nitrate or not due to the absence of nitrate. However, induction was transient and decayed shortly after its maximum expression. Induction in the Δ*fnr* mutant was equally fast but to a much higher level (65-fold). Intriguingly, this gene was not induced at all in the Δ*narG* mutant. This expression pattern is difficult to explain but one hypothesis is that this gene could at least temporarily be induced by anoxic conditions in an Fnr-independent way, but that Fnr could be actually repressing its expression when becoming active.

### Identification of Fnr DNA binding sites

**Figure 8:**
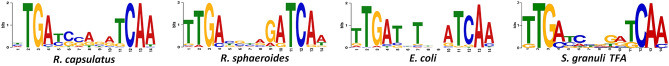
Consensus sequence for the Fnr box of TFA. Consensus sequence logo obtained for the Fnr target promoters in TFA compared with the previously defined Fnr consensus target sequences in *R. capsulatus*, *R. sphaeroides* (two α-*Proteobacteria*) and *E. coli*^39^*.* The TFA logo was obtained with the bioinformatic tool MEME^39^ using as input the putative promoter sequences of genes upregulated more than threefold in the Δ*narG* mutant MPO253 with respect to the Δ*fnrN*Δ*fixK* mutant MPO252.

Transcription initiation sites in the TFA genome were previously detected by RNA-Seq using samples treated with terminal exonuclease^13^, which together with gene separation and orientation allowed to establish putative operons of the TFA genome. In order to identify an Fnr box to which the TFA Fnr proteins would bind to regulate the expression of their target genes, we initially selected anaerobically induced genes expressed more than threefold in the Δ*narG* MPO253 mutant as compared to the Δ*fnrN*Δ*fixK* MPO252 double mutant (Suppl. Table S1, tab 3), and analysed the upstream regions of the first genes of the operons where the selected genes were comprised, using the bioinformatic tool MEME, which looks for conserved patterns among the provided sequences^39^. We then looked for the sequence pattern obtained in all the genes analysed in the first instance, using the bioinformatic tool FIMO. The genes that showed this consensus sequence were once again analysed using MEME to obtain a more refined Fnr box for TFA. Although there was a small variability in some of the bases, the most common consensus sequence for the Fnr box in TFA was TTGAc/t-N_4_-g/aTCAA (Fig. 8).

A search for the consensus sequences was then performed using FIMO in the upstream regions of the operons that showed at least threefold upregulation in the Δ*narG* mutant compared to the Δ*fnr* mutant of at least one of their genes, even though the operon had not shown substantial anaerobic induction. This identified 12 operons bearing at least one Fnr box upstream (Table 1, top part). Operon SGRAN_3845-3856, comprising the genes for nitrate respiration, and operon SGRAN_4065-4071 appear to have an internal Fnr site upstream SGRAN_3856 and SGRAN_4067 respectively. All genes in category 1 are comprised in one of these operons, except for SGRAN_3352 and SGRAN_0601.

**Table 1:** Fnr boxes in regulated operons.

SGRAN number	Gene name	Centre	Description	Operon
0602	*cybB*	− 42.5	Cytochrome *b*_*561*_	Monocistronic
2449	*ccoN*	− 41.5	Cytochrome *c* oxidase polypeptide I-like protein	SGRAN_2449-2458
2458	*hemN2*	− 42.5	O_2_-independent Coproporphyrinogen-III oxidase	Monocistronic
3353	*uspA*	− 41.5	Universal stress protein UspA	Monocistronic
3590	*nifA5*	− 93.5	σ^N^-dependent transcriptional activator	Monocistronic
3802	EBMC1_01250	− 41.5	Cupin	SGRAN_3802-3800
3843	BAY1663_04176	− 41.5	Uncharacterised protein	Monocistronic
3845	*narU*^a^	− 42.5	Nitrate/nitrite transporter NarU	SGRAN_3845-3856
3858	*yhbT* (*ubiT*)	− 40.5	Anaerobic ubiquinone biosynthesis	SGRAN_3858-3860
3862	*nrdZ*	− 41.5	Ribonucleotide reductase	Monocistronic
3916	EBMC1_03969	− 40.5	Flavin containing monooxygenase	Monocistronic
4065	*acdA_2*^b^	− 41.5	Acyl-CoA dehydrogenase	SGRAN_4065-4071
0222	*moeB*	ND	Molybdenum cofactor biosynthesis protein MoeB	SGRAN_0221-0222
1450	AI27_13850	− 51.5	Outer membrane protein	Monocistronic
1553	*gltD*	− 328	Glutamate synthase subunit	Monocistronic
2754	*fabZ*	ND	3-hydroxyacyl-[acyl-carrier-protein] dehydratase	SGRAN_2756-2754
2992	*regB*	− 43.5	Histidine kinase	SGRAN_ 2997–2991
3861	*fixK*	− 60.5	Putative Fnr protein	Monocistronic

The gene name of the first gene of the operon and its description is indicated.

ND: TSS not identified.

^a^Upstream of *narU* there are 3 Fnr boxes. There is an additional internal site upstream of *moeA* (SGRAN_3856).

^b^There is an additional internal site upstream SGRAN_4067.

There were 3 monocistronic operons, *cybB*, *hemN2*, and *nifA5*, with putative Fnr boxes whose genes had not been ascribed to category 1. This is because they showed poor upregulation in anaerobiosis (< threefold). In addition, *nifA5* was not downregulated in the Δ*fnr* mutant.

The Fnr boxes of most of these genes (*cybB*, *ccoN*, *hemN2*, *uspA*, SGRAN_3802, SGRAN_3843, *narU*, *yhbT*, *nrdZ*, SGRAN_3916 and *acdA_2*) were centred at − 41.5 with respect to the transcription initiation sites, with slight displacements in some of them, thus overlapping the − 35 region. Therefore, they belong to class II Fnr-activated promoters^19,40^. On the other hand, the one at *nifA5* was located centred at − 93.5, a position found at Class I promoters.

Upstream of *ccoN* and divergently transcribed, is *ompW,* which in *E. coli* codes for a porine involved in metabolic transitions in anaerobiosis and that is regulated by Fnr^41^. This gene was also induced in anaerobic conditions, although it was not sufficiently downregulated in Δ*fnr* (2.75-fold downregulation) to pass the cutoff. It could be that the Fnr site upstream of *ccoN* could also serve as an activation site for *ompW*. Two additional Fnr sites have been found upstream *narU*. They might be activator sites for the divergently transcribed *ftrB*, encoding an Fnr-like transcriptional activator similar to NarR, reported to be involved in regulating expression of the respiratory nitrate reductase in response to nitrate/nitrite^42^, and that, similarly to *ompW*, is regulated by anaerobiosis although not sufficiently downregulated in Δ*fnr* (2.97-fold downregulation).

An additional search was performed in the upstream regions of operons that were downregulated at least threefold in the Δ*fnr* mutant as compared to the WT, regardless their expression in the Δ*narG* mutant and their anaerobic induction. This resulted in the identification of 6 additional operons bearing putative Fnr boxes upstream (Table 1, bottom part).

The SGRAN_0221-0222 and SGRAN_ 2997-2991 (*cyoABCDsurF1regBA*) operons were ascribed to category 2 and code for proteins involved in Mo cofactor biosynthesis and an alternative electron transfer chain, respectively. In both cases the Fnr binding sites detected by FIMO were inside the operons, as if internal promoters were directly activated by Fnr proteins. Manual inspection did not reveal Fnr sites upstream of the first genes of these operons. However, all genes of these two operons were strongly induced in anaerobiosis, thus suggesting that their anaerobic induction might be indirect.

The remaining genes were not sufficiently induced in anaerobiosis to be ascribed to categories 1 or 2; in fact SGRAN_1450 seemed downregulated in anaerobiosis and *gltD* showed a putative Fnr binding site very far upstream, centred at -328, thus making it an unlikely Fnr target.

The activator gene *fixK*, which apparently is directly activated by FnrN, bears an Fnr binding site centred at − 60.5, thus representing a Class I promoter.

An additional search was performed among the genes that showed at least threefold upregulation in the Δ*fnr* mutant as compared to the WT, in an attempt to detect potential Fnr repression binding sites. Potential binding sites were identified in the proximity of the 5′ ends of 19 genes. The positions relative to the initiation codons were very variable. However, only 5 of these genes were downregulated in anaerobiosis ≥ threefold and all 5 were similarly upregulated in the Δ*fnr* and in the Δ*narG* mutants, thus suggesting that they were not actually repressed by Fnr.

Finally, a search was performed at the 5′ ends of all genes encoded in the TFA genome. The search identified 96 potential Fnr binding sites additional to those previously identified. However they were barely expressed in any condition or in the mutants, which were not considered in the dRNA-Seq analysis, they had a putative Fnr box very far from their TSS, or they did not meet some of the necessary premises to be considered. One possible exception is SGRAN_1582, which bears an Fnr site centred at − 29.5, thus overlapping the − 35 promoter region.

## Discussion

DNA sequence analysis of the strain TFA genome revealed the presence of two putative *fnr-*like genes annotated as *fnrN* and *fixK*. Comparative analysis of both gene products to homologous Fnr proteins shows that they display the conserved cysteine motif involved in binding of the oxygen-labile [4Fe–4S]^2+^cluster of the Fnr group of proteins, though with a slightly modified arrangement characteristic of the FnrN subgroup of the CRP-FNR superfamily of transcriptional regulators. This displacement is representative of *α-Proteobacteria* such us *Rhodobacterales, Rhodospirillales*, and *Sphingomonadales*^23^. Both have an Fnr function since both complement *E. coli fnr* and TFA double mutant. However, FixK cannot completely supply FnrN function, even when transcribed from the strong *tac* promoter, especially in MM. Also, it cannot complement the *E. coli fnr* mutant as efficiently as FnrN. This difference in efficiency cannot be explained by differences in affinity for the Fnr boxes since the FixK HTH is even more similar to *E. coli* Fnr HTH than FnrN HTH. We therefore should conclude that FixK homodimers are less functional than FnrN homodimers. Transcription of *fixK* is activated under nitrate respiring conditions apparently by FnrN. Then, what physiological meaning could have that FnrN induces expression of a less efficient activator as FixK?

An obvious paralelism is observed in the Global Stress Response of this bacterium, controlled by two extracytoplasmic function sigma factors EcfGs, in which *ecfG1*, whose expression is activated by the constitutively produced EcfG2, just plays an auxiliary role in modulating the global stress response^43^. In this case, a similar auxiliary role of FixK in the global Fnr-mediated activation control could be exerted by competing for Fnr target sites or by forming FnrN-FixK inefficient heterodimers, which would result in a baroque but functional way of auto-modulating Fnr activity under anaerobic conditions. An alternative possibility that cannot be ruled out is that FrnN-FixK heterodimers are actually functional, thus resulting in an increase of the amount of active Fnr dimers in response to anaerobiosis.

Since the Δ*fnr* double mutant cannot grow anaerobically we attempted to identify genes regulated by Fnr by performing dRNA-Seq analyses comparing anaerobic expression in the regulatory mutant and in the Δ*narG* that is also unable to grow anaerobically. These analyses together with identification of Fnr boxes in operons putatively regulated by Fnr have been useful to define a minimal Fnr regulon in TFA, even though most of the anaerobically induced and Fnr activated genes were expressed to very low levels in the structural Δ*narG* mutant. It appears that other Fnr regulons of *α-Proteobacteria* are much larger^22,44,45^. It might be that the Fnr-mediated response to anaerobiosis in *Sphingopyxis granuli* is limited to fewer genes than in *Rhodobacter*. However, this is difficult to ascertain because we have been more strict in establishing the cutoff for regulation.

The most obviously Fnr-regulated genes belong to category 1, although some of the ones in category 2 could also be directly regulated by Fnr. Expression pattern of genes in category 2 suggests that their induction is strongly dependent on nitrate respiration, which prevents any interpretation about whether these genes could be regulated by Fnr or not since the Fnr-activated expression of some of these genes would be masked by the need of anaerobic growth for induction. Actually, a few operons such as *fixK* itself, *moeB*, *regBA* and *ubiTUV* appear to be directly activated by Fnr, since they bear an Fnr binding site at the right position.

On the other hand, other genes of category 2 might be indirectly regulated by Fnr or by other regulators in response to changes in environmental conditions associated to anaerobic growth on nitrate. Regarding the first possibility, the Fnr proteins appear to activate expression of the regulatory gene *nifA5* and the *regBA* operon. The *regBA* tandem codes for a redox-responding two-component regulatory system involved in coordinating adaptation of bacterial respiration to anoxic conditions in different α- and γ-proteobacterial species^18,46,47^. Expression of numerous genes have been shown to be regulated by both FnrL and RegA in *Rhodobacter*^22^. In strain TFA, the induced RegBA products could, in turn, activate the expression of the whole *cyoABCDsurF1regBA* operon, thus resulting in a strong auto-regulated increase of the RegBA synthesis, and, eventually, induction other operons in a second tier of regulation.

Besides its well characterised activating function at two Classes of promoters, Fnr is also known to directly repress genes differentially expressed in anaerobiosis^44,48^. However, in TFA, the great majority of genes that were potentially repressed by Fnr were upregulated in both *fnr* and *narG* mutants. Besides, just a few Fnr binding sites have been found within the vicinity of these genes and their relative positions do not suggest that they might be directly repressed by Fnr. One possible exception is SGRAN-1582, which bears an fnr site centred at − 29,5, thus overlapping the − 35 promoter region. Since it codes for a regulator and is divergent to an operon with the same expression pattern, which codes for enzymes of an aerobic degradation pathway, it is tempting to speculate that Fnr may repress expression of this biodegradation pathway indirectly through repressing expression of its activator.

Genes involved in stress detoxification are not regulated by anaerobiosis or Fnr. This is not surprising considering their function and the fact that at least some of them were slowly induced in response to anaerobiosis^17^. They belong to categories 2 and 3, which suggest different forms of regulation of these genes. Those belonging to category 2 do not appear to be directly upregulated by anaerobiosis or Fnr (they lack Fnr boxes). Rather, they appear to respond to the accumulation of toxic products associated to nitrate respiration metabolism or to lesions generated by them such as accumulation of nitrite, which is toxic, indirectly mutagenic^49^ and shown to stop anaerobic growth of strain TFA^17^, or nitric oxide, apparently generated by the nitrate reductase itself^17,50^, which generates nitrosative stress even in anaerobiosis that results, among other effects, in damage of Fe-S clusters of proteins^33^. Those belonging to category 3 appear to be genes responding to an energy limitation stress due to anaerobic respiration in the WT strain, which is intensified in the absence of respiration in the WT in the absence of nitrate or in the mutants unable to respire nitrate. However, this response appears to be specific of anaerobiosis because these genes are not induced under other energy limiting conditions such as carbon starvation in aerobiosis, which induces the General Stress Response of this bacterium^43^. One possibility is that they respond to a high of NADH/NAD^+^ ratio but the nature of the signal or the response mechanism remains uncharacterised.

Also, it is worth noting that expression of a substantial number of anaerobically regulated genes were not affected in Δ*fnr* or Δ*narG* mutants. One explanation is that their expression is regulated by other regulators completely independent of Fnr. However, the implication of *regBA* in regulating expression of these genes could also be possible. Although *regBA* transcription is activated by Fnr, there is a substantial *regBA* expression even in aerobiosis (its TSS was very evident in aerobiosis^13^), thus suggesting that *regBA* might regulate a number of genes in response to the redox status even though its transcription were not activated by Fnr.

Finally, *Sphingopyxis* is a bacterial genus generally regarded as strictly aerobic. However, strain TFA is able to grow using nitrate as the alternative electron acceptor producing nitrite and nitric oxide, which are toxic. This work has characterised the regulation of the genes differentially expressed in anaerobiosis, characterised the Fnr regulon and showed that expression of many other genes is not directly regulated by Fnr but is indirectly responding to anaerobiosis or to signals or metabolites accumulated during this partial respiration of nitrate, which contributes to our understanding of the physiological changes that this *Sphingopyxis* bacterium has to make in order to thrive in anaerobic environments.

## Methods

### Bacterial strains, plasmids, primers and growth conditions

*Escherichia coli* strain M182^51^ and its Δ*fnr* derivative mutant strain JRG6348^52^ were used for functional complementation assays. *E. coli* strain DH5α^53^ and DH5α λpir were used for cloning. Previously described plasmids used are pIZ1016^54^, pGS2350^52^, pSW-I^55^ and pEMG^56^. *S. granuli* strains used in this study were wild type strain TFA, MPO250 mutant with an almost complete deletion of *fnrN* gene, MPO251 with an almost complete deletion of *fixK* gene, MPO252 double mutant with an almost complete deletion of both *fnrN* and *fixK* genes and MPO253 mutant with an almost complete deletion of the *narG* gene.

MPO0253 was previously constructed^17^. MPO250, MPO251 and MPO252 mutants were constructed using a previously described method^56^ with slight modifications^17^. Regions 1 kb upstream and downstream of the *fnrN* and *fixK* genes were amplified by PCR with the primers fnrNF1F, fnrNF1R, fnrNF2F, fnrNF2R, fixKF1F, fixKF1R, fixKF2F, fixKF2R respectively. *fnrN* and *fixK* flanking regions were cloned in pEMG using the restriction enzymes EcoRI and BamHI for the upstream regions and BamHI and XbaI for the downstream regions. The constructions were checked by sequencing. To construct the single mutants MPO250 and MPO251 the generated plasmids containing the 688 bp deletion of *fnrN* and the 682 bp deletion of *fixK* were transformed separately by electroporation in *S. granuli* TFA wild type strain. Cells resistant to kanamycin, which should have the corresponding plasmid integrated in the chromosome by homologous recombination, were selected and the candidate cells checked by PCR with the primers AguBF and M13 Rv and FnrNF1F and M13 Rv for the *fnrN* deletion plasmid and with the primers KmFw-pk18 and KmRv-pk18 and FfixKCompF and M13Rv for *fixK* deletion plasmid. A positive candidate was electroporated with pSW-I plasmid and candidates resistant to ampicillin but sensitive to kanamycin, which should have undergone the second recombination event, were selected. Candidates were checked by PCR with the primers pSW-F and pSW-R. Positive candidates were grown for several generations without ampicillin and ampicillin sensitive candidates that have lost the pSW-I plasmid were selected and the mutation checked by PCR, with the primers AguBF and FnrNF2R for MPO250 mutant and FFixKcompFw and fixKF2R for MPO251 mutant, and by Southern blot. For MPO250 mutant, the probe used for the Southern blot was the PCR product of the primers fnrNF2F and fnrNF2R and the genomic DNA was digested with BamHI, NcoI and EcoRV + SmaI. For MPO251 mutant the probe used was the PCR product of the primers fixKF2F and fixKF2R and the genomic DNA was digested with AatII, PvuII and NdeI + SspI. To construct the double MPO252 mutant the single mutant MPO251 was electroporated with the pEMG plasmid containing the flanking regions of *fnrN* and the same protocol as with the single mutants was followed, checking the candidates by PCR with the primers AguBF and FnrNF2R and by Southern blot using the same probe and restriction sites as for the MPO250 mutant.

For the construction of pMPO704 plasmid, *fnrN* gene was amplified using fnrNHindIIIF and fnrNXbaIR primers and cloned in pIZ1016 plasmid using the restriction enzymes HindIII and XbaI. For the construction of pMPO705 plasmid, *fixK* gene was amplified using fixKPstIF-2 and fixKXbaIR-2 primers and cloned in pIZ1016 plasmid using the restriction enzymes PstI and XbaI. pMPO704 and pMPO705 plasmids were introduced in TFA WT, MPO250, MPO251, MPO252 by electroporation and positive candidates were selected by antibiotic resistance and checked by PCR with the oligos fnrNHindIIIF and F9 for pMPO704 and fixKPstIF-2 and F9 for pMPO705. pIZ1016, pMPO704 and pMPO705 plasmids were introduced in JRG6348 by TSS method^57^ and positive candidates selected by antibiotic resistance.

Rich medium for *S. granuli* strains was MML^9^ whilst that for *E. coli* strains was LB medium^58^. *S. granuli* was grown at 30 °C whilst *E coli* at 37 °C, both in liquid culture and solid plates. Antibiotic concentrations used for TFA were streptomycin 50 μg/mL, kanamycin 20 μg/mL, gentamicin 10 μg/mL and ampicillin 5 μg/mL. Antibiotic concentrations used for *E. coli* were chloramphenicol 30 μg/mL, ampicillin 100 μg/mL and gentamycin 10 μg/mL.

Primers used in this work are listed in Supplementary Table S2.

### Nitrite and nitrate determination

Nitrite and nitrate concentrations in the growth media were measured as described previously^14,59^. For the complete protocol, see Supplementary material.

### Functional complementation of *E. coli* JRG6348 strain

For functional complementation, growth curves of JRG6348 strain with TFA *fnrN* or *fixK* genes, cloned in pMPO704 and pMPO705 plasmids, respectively, were performed in mineral medium^51^ with casamino acids 0.5 g/L, the non-fermentable carbon source glycerol 40 mM and sodium nitrate 20 mM. The M182 strain with the empty plasmid pIZ1016 and the Δ*fnr* mutant strain JRG6348 complemented with the empty plasmid (pIZ1016), with an *E. coli fnr* (pGS2350), and with TFA *fnrN* (pMPO704) and *fixK* (pMPO705) were aerobically grown in liquid cultures overnight at 37 °C in mineral medium with casaminoacids and glycerol as described above without nitrate and then diluted to an OD_600_ of 0.1 in the same medium adding 20 mM of sodium nitrate, transferred into standing stoppered bottles filled to the top and grown at 37 °C. Replicates with IPTG 1 mM of JRG6348 pMPO704 and pMPO705 as an inducer of the expression from the *tac* promoter were also carried and each experiment was repeated twice. OD_600_ of the cultures was measured over time.

### Functional complementation of MPO250 and MPO252 mutants

Functional complementation curves of MPO250 and MPO252 mutant strains with pMPO704 and pMPO705 were performed both in MML rich medium and in mineral medium with β-hydroxybutyrate as the carbon and energy source. The strains were grown overnight in MML or for 2 days in mineral medium, and then respectively diluted to an OD_600_ of 0.1 in the same medium but containing sodium nitrate 20 mM, transferred into standing stoppered bottles filled to the top and grown at 30 °C. Replicates with IPTG 1 mM of MPO250 with pMPO704, MPO252 with pMPO704 and MPO252 with pMPO705 were also carried and each experiment was repeated twice. OD_600_ of the cultures was measured over time.

### Motif search and sequence analysis

450 bp sequences upstream the start codon of genes more than threefold downregulated in MPO252 compared to MPO253 were subjected to motif search using the online tool MEME. The significant parameters considered in the analysis were: motif occurrences: 0 or 1; number of different motifs: 5; minimum and maximum motif width: 13–15 (finally adjusted to 14 bp); searching only in the given strand and eventually searching only for palindromic patterns. Consensus sequence retrieved by MEME^39^ was thrown back to the pool of sequences using FIMO in order to detect low quality matches. Resulting sequences were manually inspected according to the distance of the putative consensus to possible transcription start sites previously described in this strain^60^ or to the respective gene start codon. Also, the putative operon organization of the regulated genes was analysed according to the expression data shown in this work and in previously reported data^60^ by visualizing them using the Integrated Genome Browser (IGB). The Fnr consensus logo was generated using MEME^39^ after the manual curation of the putative Fnr target promoters.

### dRNA-seq sample preparation and analysis

For high-throughput RNA sequencing, strains were grown in mineral medium with 40 mM β-hydroxybutyrate as the only carbon and energy source at 30 ºC. In the case of TFA strain, it was grown anaerobically with sodium nitrate 20 mM as terminal electron acceptor from an initial optical density at 600 nm of 0.1 to 0.7–0.8. Regarding MPO252 and MPO253, they were grown aerobically from optical density at 600 nm of 0.1 to 0.7–0.8 and then incubated for 6 h in anoxic conditions with sodium nitrate 20 mM. Samples of three biological replicates of each strain were taken and their RNA extracted and mixed. Samples were sent to the company ASCIDEA for cDNA library preparation and high-throughput sequencing using an Illumina HiSeq2000 machine. Normalization and differential expression analyses were performed by ASCIDEA. Detail information is provided in the Supplementary material.

### RT-qPCRs assays

To validate the RNA-Seq analysis and to study the expression levels of selected genes along time in different conditions, we performed RT-qPCRs in these conditions.

For the WT and MPO250 strains in anaerobic conditions with nitrate in mineral medium with β-hydroxybutyrate, samples were prepared as described before^17^: cells were grown aerobically in mineral medium until the exponential phase and then diluted to an initial optical density of about 0.1 in the same medium. After one hour of aerobic incubation, the initial time 0 was taken, sodium nitrate 20 mM was added to the cultures which were then transferred into standing stoppered bottles filled to the top and incubated under anaerobiosis during the rest of the assay, taking samples periodically for RNA purification. For the wild type strain in anaerobic conditions without nitrate and MPO252 and MPO253 mutants in anaerobic conditions with nitrate, cells were grown in mineral medium in aerobic conditions until an optical density of about 0.7. Then, time 0 was taken and the cultures were incubated under anaerobic conditions during the rest of the assay, adding a 20 mM concentration of nitrate in the case of the mutants, and taking samples periodically for RNA purification. Three independent biological replicates of each growth condition were treated for RNA purification.

Total RNA extraction was carried out as described above and in previous works^17,61^. DNase I treatment was performed with a DNA-free kit (Ambion). The samples were purified using RNAeasy columns (Quiagen) and RNA quality was confirmed by non-denaturing agarose gel electrophoresis. The absence of contaminating DNA was then confirmed by PCR amplification. Equal amounts of RNA obtained from three independent biological replicates of each growth condition were mixed and used in the gene expression analysis. Retro-transcription of these RNA mixes was performed using the High-Capacity cDNA Archive Kit (Applied Biosystems), with random hexamers as primers to generate cDNAs. The resulting cDNA samples were amplified by RT-qPCR using 0.3 mM of each primer as previously described^17,62^. The results are the average of 3–4 technical replicas. In all conditions fold change induction with respect to time 0 is represented.

## Supplementary information

Supplementary Information 1.

Supplementary Information 2.

## Data Availability

All data generated or analysed in this study are included in this article.
