# Supplementary material for: Identification of two fnr genes and characterisation of their role in the anaerobic switch in Sphingopyxis granuli strain TFA
Source: Sci Rep. 2020 Dec 3;10:21019. doi: 10.1038/s41598-020-77927-w (PMC7713065; doi:10.1038/s41598-020-77927-w)
Supplement: Supplementary file 2 — Supplementary Information 2. [file 41598_2020_77927_MOESM2_ESM.pdf]

SUPPLEMENTARY INFORMATION

Identification of two *fnr* genes and characterisation of their role in the anaerobic switch in *Sphingopyxis granuli* strain TFA

Yolanda Elisabet González-Flores, Rubén de Dios, Francisca Reyes-Ramírez* and Eduardo Santero.

Centro Andaluz de Biología del Desarrollo, Universidad Pablo de Olavide/Consejo Superior de Investigaciones Científicas/Junta de Andalucía and Departamento de Biología Molecular e Ingeniería Bioquímica, Universidad Pablo de Olavide.

***Corresponding author: Francisca Reyes-Ramírez**

Address: Centro Andaluz de Biología del Desarrollo. Universidad Pablo de Olavide-CSIC-Junta de Andalucía. Carretera de Utrera, Km. 1 41013 Sevilla, Spain.

Telephone: +34-954348644. Fax: +34-954349376. E-mail: freyram@upo.es

Sample preparation for high-throughput RNA sequencing

For all growth conditions, TFA was previously grown aerobically at 30°C in mineral medium[1] containing 40 mM β-hydroxybutyrate as the only carbon and energy source to the exponential phase (optical density at 600 nm 0.7-0.8). In the case of the WT strain growing in anaerobic conditions samples were prepared as described before[2]. Briefly, cells were diluted to an initial optical density of about 0.1 into a fresh medium with 20 mM sodium nitrate as final electron acceptor, the appropriate cultures were transferred into standing stoppered bottles filled to the top and cultures were grown at 30°C until they reached a final optical density of 0.7-0.8, when samples for RNA extraction were taken. In the case of MPO252 and MPO253 mutants, cells were also diluted into a fresh medium to an initial optical density of about 0.1 and they were grown in minimal medium in aerobic conditions until an optical density of about 0.7-0.8. Then, sodium nitrate 20 mM was added, the cultures were transferred into standing stoppered bottles filled to the top and cultures were incubated at 30°C during 6 hours, when samples for RNA extraction were taken.

Total RNA extraction was carried out as previously described[2,3] (see Methods). RNA samples were sent to the ASCIDEA Headquarters, Barcelona (Spain) (http://www.ascidea.com/lifesciences-services.html) for library preparation and RNA sequencing. The cDNA libraries were sequenced using an Illumina HiSeq2000 machine.

Bioinformatic analysis

The normalization and the analyses of the dRNA-seq results were performed by ASCIDEA. Briefly, FastQC software FASTX-Toolkit (http://hannonlab.cshl.edu/fastx_toolkit/index.html) was used to check quality of the reads obtained by HiSeq2000 sequencing and ASCIDEA specific perl scripts were run as low quality region filters. Adaptors and low quality bases at the ends of sequences and reads with undetermined bases or with 80% of their bases with less than 20% quality score were trimmed. Reads that passed these filters were mapped using [4] to generate read alignments using the *Sphingopyxis granuli* strain TFA genome (RefSeq:NZ_CP012199.1)[5]. Differential transcript expression was then computed using DESeq2.

Nitrite and nitrate determination

Nitrite and nitrate concentrations in the growth media were measured as described previously[5,6]. Briefly, cultures were first centrifuged and filtered. For nitrite determination, 300 µl of the appropriate dilutions of each filtered medium sample were mixed with 300 µl of a saturated solution of sulfanilic acid prepared in 20 % (v/v) of HCl and 300 µl of aqueous solution of 0.2 % (w/v) N-(1-naphthyl)-ethylendiamine dihydrochloride, incubated for 15 minutes at room temperature, and absorbance at 540 nm was measured. Nitrite concentration was determined using a standard curve prepared with sodium nitrite. For nitrate determination, nitrite was first removed from the medium by the addition of amidosulfonic acid to a final concentration of 1 % (w/v) and overnight incubation. Then 100 µl of the appropriate dilutions of each filtered medium sample were mixed with 800 µl of an H_2SO_4:H_3PO_4 solution (1:1 in volume) and 100 µl of a fresh solution of 2, 6-dimethylphenol (0.12 %, w/v, in concentrated acetic acid), incubated for 20 minutes at room temperature, and absorbance at 324 nm was measured. Nitrate concentration was determined using a standard curve prepared with sodium nitrate.

Supplementary table S1: (provided as excel file). dRNA-seq analyses in the double Δ*fnr* mutant and Δ*narG* mutant strains and organization of the differentially expressed genes in different categories.

Supplementary table S2. Primers used in this work.

Name	Sequence 5' --> 3'	Function
fnrNF1F	CCCATCACGAATTCGCCATGCC	Forward primer for the amplification of the upstream region of *fnrN* gene in TFA
fnrNF1R	CATCGCAGGGATCCATGATTTAAAG	Reverse primer for the amplification of the upstream region of *fnrN* gene in TFA
fnrNF2F	CACTCGCCGGATCCTCAGAAGCG	Forward primer for the amplification of the downstream region of *fnrN* gene in TFA
fnrNF2R	GACGATTCTCTAGAAGACGGTCG	Reverse primer for the amplification of the downstream region of *fnrN* gene in TFA
fixKF1F	GAAGCGCGAATTCTATGCCCG	Forward primer for the amplification of the upstream region of *fixK* gene in TFA
fixKF1R	CTTCGTCAGGATCCGCTTTCCCG	Reverse primer for the amplification of the upstream region of *fixK* gene in TFA
fixKF2F	CGAGATTCTGGATCCACGCGGG	Forward primer for the amplification of the downstream region of *fixK* gene in TFA
fixKF2R	GGCACCATCTAGAAGGCCGCC	Reverse primer for the amplification of the downstream region of *fixK* gene in TFA
AguBF	GCAATTTGTCCAAGGCGTTCGG	Primer that anneals in the gene upstream *fnrN* used for checkings
M13Rv	TCACACAGGAAACAGCTATGAC	Primer that anneals in pEMG and its derivatives used for checkings
KmFw-pk18	GATTGAACAAGATGGATTGC	Forward primer that amplifies the Km gene of pEMG and its derivatives used for checkings
KmRv-pk18	CGTCAAGAAGGCGATAGAAGG	Reverse primer that amplifies the Km gene of pEMG and its derivatives used for checkings
pSW-F	GGACGCTTCGCTGAAAACTA	Forward primer that amplifies a fragment of pSW-I used to check the presence of this plasmid
pSW-R	AACGTCGTGACTGGGAAAAC	Reverse primer that amplifies a fragment of pSW-I used to check the presence of this plasmid
FFixKcompFw	CATCGCTATTGGCCGGCCCG	Forward primer than anneals in the upstream flanking region of fixK gene used for checkings
fnrNHindIIIF	GATGGAAGCTTTAAATCATGAATTC	Forward primer to amplify fnrN gene of TFA for cloning
fnrNXbaIR	CTGCGCTCTAGATCAGCCGGCG	Reverse primer to amplify fnrN gene of TFA for cloning
fixKPstIF-2	ATCGGACTGCAGTTTGCGATTGC	Forward primer to amplify fixK gene of TFA for cloning
fixKXbaIR-2	GCGGAGCGTCTAGAGGGGCCTG	Reverse primer to amplify fixK gene of TFA for cloning
F9	CTATAGGGCGAATTGGAGCTC	Reverse primer that anneals in pIZ1016 and its derivatives used for checkings
fixK qPCR Fw	CGAGACGCTGGCCGATTTC	Forward primer for *fixK* gene RT-qPCR
fixK qPCR Rv	TCCGACAGGATCGCGTAAAG	Reverse primer for *fixK* gene RT-qPCR
fnrN qPCR Fw	GACTCCGTCGAGGTTGAG	Forward primer for *fnrN* gene RT-qPCR
fnrN qPCR Rv	GTACCGGTGGACAATTTGAG	Reverse primer for *fnrN* gene RT-qPCR
narG qPCR Fw	GCTGACCTGGTTCGTCTAC	Forward primer for *narG* gene RT-qPCR
narG qPCR Rv	AGCTGGCTCATGCGGAAAC	Reverse primer for *narG* gene RT-qPCR
ccoH qPCR Fw	GTTTCTTCGGCACGATCATC	Forward primer for *ccoH* gene RT-qPCR
ccoH qPCR Rv	CATCGTCGAGCCACTTGTTG	Reverse primer for *ccoH* gene RT-qPCR
nrdZ qPCR Fw	CTGCGAGCGGGAGTTTCTG	Forward primer for *nrdZ* gene RT-qPCR
nrdZ qPCR Rv	GTCGAGATCGTCGGGAATC	Reverse primer for *nrdZ* gene RT-qPCR
nnrS2 qPCR Fw	CGCCTACCGCCTGTTCTTC	Forward primer for *nnrS2* gene RT-qPCR
nnrS2 qPCR Rv	ACAGGGTGACGACGATGAC	Reverse primer for *nnrS2* gene RT-qPCR
aox qPCR Fw	CGCGGTCGTGCTCGAAAC	Forward primer for *aox* gene RT-qPCR
aox qPCR Rv	GTCTTGATCCAGCCCTTGTC	Reverse primer for *aox* gene RT-qPCR
ubiU qPCR Fw	GGCAGCCCGGAGCTCATC	Forward primer for *ubiU* gene RT-qPCR
ubiU qPCR Rv	TGAAGTTGAGGCCGGAGAAG	Reverse primer for *ubiU* gene RT-qPCR
0596 qPCR Fw	GCAGATGGGCAAGCCTTTG	Forward primer for SGRAN_0596 gene RT-qPCR
0596 qPCR Rv	CGGCAGCCTTGATCTCATAG	Reverse primer for SGRAN_0596 gene RT-qPCR
0597 qPCR Fw	CTGCCATGTTCGGATTCAAG	Forward primer for SGRAN_0597 gene RT-qPCR
0597 qPCR Rv	CGCCTTGCCGCCCTTCAG	Reverse primer for SGRAN_0597 gene RT-qPCR
SGRAN_1383 qPCR Fw	GATTCGCCCCATGTGCATTC	Forward primer for SGRAN_1383 gene RT-qPCR
SGRAN_1383 qPCR Rv	GCGGCAGCAACAGGCAATG	Reverse primer for SGRAN_1383 gene RT-qPCR

```
FnrN TFA               ----------------MNSCDACVVRNRSICAALDSVEVEALNA-IGRRRTLEPGESLIW 43
FixK TFA               ----M--TKRA-MLSKDHPCRSCDVSAEALCRALDVETLADFRN-QGGRLHLTAGQTLFH 52
FnrL R. sphaeroides    ----M--T-LHEVPTILHRCGDCPIRHRAVCARCDSEELATLEQ-IKYYRSYQAGQTVIW 52
FnrL R. capsulatus     ----M--S-HDDAHPVSLQCGDCPIRHRAVCARCESSELDELEG-AKYYRSFEAGQTVIW 52
FnrN R. leguminosarum  MDVAR--S-EFFETGTPVACTSCQARHGVVCGALSKGQLRELNR-HSLRRKIEAGCEIIA 56
FixK B. diazoefficiens MKPSV--V--MIEPNGHF-CSDCAIRTSAVCSSLDAAELREFEH-LGRRVHFSSGETVFS 54
AadR R. palustris      -MPHL--AYPTTTCEGFRCETHCAVRGLAICGELGPADHEEFER-LAQHVRYGPKEALFS 56
Fnr R. etli            MDVAR--S-EVLVIGTSVACRSCQARHGVVCGALSAGQLSELGR-HSLRRKVDAGCEIIA 56
Btr B. pertussis       ---MQR-RVPL--SPDAAHCSSCMLGHVCVPVGMPANEVEKLDELVKERVRVERGKTLYE 54
AcpR Azoarcus sp.      -MQMKA-TVPITVASLKVACSQCNLVELCLPFGMSESEIDRLDELVGARRKIKRQQNLYR 58
Fnr A. aromaticum      -MQMKA-TVPITVASLKVACSQCNLVELCLPFGMSENEIDRLDELVGARRKIKRQHHLYR 58
Fnr E. coli            MIPEKRIIRRIQSGGCAIHCQDCSISQLCIPFTLNEHELDQLDNIIERKKPIQKGQTLFK 60
Anr P. aeruginosa      -MAETIKV----RALPQAHCKDCSLAPLCLPLSLTVEDMDSLDEIVKRGRPLKKGEFLFR 55
                                             *      :           :               :

FnrN TFA               EDSESVLVANVVEGVLKLSTGTEDGREQIVGVVYPSDFIGRPFGAT--TPHSVTAMTEAK 101
FixK TFA               QGDPADCVFSLTSGVVKLYAILSDGRRQIVAFLFPGDFVGFETQQS--HGFAAEAIGDTT 110
FnrL R. sphaeroides    SGDKMDFVASVVTGIATLTQTMEDGRRQMVGLLLPSDFVGRPGRQT--VAYDVTATTDLL 110
FnrL R. capsulatus     SGDKMDFVASVVSGIATLTQTLEDGRTQMVGLLLPSDFVGRPGRDR--AAYNVTATTDIL 110
FnrN R. leguminosarum  QGSESSFYSNIMRGVMKLCKVMPDGRQQIVGLQFAPDFVGRPFVRE--STLSAEAATDSE 114
FixK B. diazoefficiens EEDITTSFYNVLEGVMRLYKLLPDGRRQIVGFALPGDFLGMNLSGR--HNFSADAIGAVT 112
AadR R. palustris      EDEVADSVYSLIEGIARLYKLLPDGRRQIIGFALPGDFLGMAPGNR--YSFSADSIGGVT 114
Fnr R. etli            QGSENSFYSNITRGVVKLCKVMSDGRQQIVGLQFAPDFVGRPFVRE--STLSAEAATDAE 114
Btr B. pertussis       LDDPLDAVYGVRFGSLKTQLEDSSGQLQITGFHLPGEIVGLDGMIESKHVSSAVALEDSE 114
AcpR Azoarcus sp.      AGDPFEAIYAIRAGSFKTDVLLEDGREQVTGFQMTGEMLGLDGISTETHSCNAIALEDSE 118
Fnr A. aromaticum      AGDPFEAIYAIRAGSFKTNVLLEDGREQVTGFQMTGEMLGLDGISTELHSCNAIALEDSE 118
Fnr E. coli            AGDELKSLYAIRSGTIKSYTITEQGDEQITGFHLAGDLVGFDAIGSGHHPSFAQALETSM 120
Anr P. aeruginosa      QGDPFGSVFAVRSGALKTFSITDAGEEQITGFHLPSELVGLSGMDTETYPVSAQALETTS 115
                         .       :  *          *  *: ..    :::*            . :

FnrN TFA               VCVFSRTDFDGFASRHPALEHKLLQRTLTELDRTRRWMLLLGRKNAEERVATFLLDMSER 161
FixK TFA               LCRVLKRRFEWFVDHYPALAEARYRRATAELAIAQERMVTLGRQTAAERLAGFLSDIQRR 170
FnrL R. sphaeroides    MCCFRRKPFEEMMQKTPHVGQRLLEMTLDELDAAREWMLLLGRKTAREKIASLLAIIARR 170
FnrL R. capsulatus     MCCFRRKPFEEMMERTPHIAQRLLQMTLDELDAAREWMLLLGRKTAREKIASLLSIVARR 170
FnrN R. leguminosarum  ICVFPRNLLDRMISETPELQRSLHDQALKELDAAREWMLTLGRRTAEEKVASLLHLIATH 174
FixK B. diazoefficiens VCQFAKAPFGRFIEERPQLLRRINELAIRELSQARDHMVLLGRRSADEKVAAFLLGWRER 172
AadR R. palustris      VCKFFRGPFLRFIENRPQMLLRMNDFATRELSLAQDQMLLLGRRSAEEKVAAFLVGWRDR 174
Fnr R. etli            ICVFPRNLLERMILETRELQRSLHAQALNELDAAREWMLTLGRRTAEEKVASLLHLITAH 174
Btr B. pertussis       VCVIRLPEIDRVSTQLPSLQQQFRRLMSREITRSHQMLATVGAMRSEQRLAAFLLNLSQR 174
AcpR Azoarcus sp.      VCVIAYSKLEELSRVVEGLQLQFHKVMSREIVRDHGVMTLLGSMRAEERLAAFLLNMSQR 178
Fnr A. aromaticum      VCVIAYGKLEELSRVVEGLQHQFHKVMSREIVRDHGVMTLLGSMRAEERLAAFLLNMSQR 178
Fnr E. coli            VCEIPFETLDDLSGKMPNLRQQMMRLMSGEIKGDQDMILLLSKKNAEERLAAFIYNLSRR 180
Anr P. aeruginosa      VCEIPFERLDELSEQLPQLRRQLMRLMSREIRDDQQMMLLLSKKTADERIATFLVNLSAR 175
                       :* .    :  .      :          *:   :  :  :.   : :::* ::     :

FnrN TFA               LVDPGCDTPDQPLDRFDLPFSRQQVGDILGLTIETVSRQFTKLKRDGIVDLPSRRAVVIR 221
FixK TFA               AGFRG----RDGANLVPLPMSRGDIADYLGLTKETVSRELTNLRKARVIRSHSLTLIEIL 226
FnrL R. sphaeroides    DAALKLRES-NGPMTFDLPLTREEMADYLGLTLETVSRQVSALKRDGVIALEGKRHVIVT 229
FnrL R. capsulatus     DASIRHRKM-RGRLSFDLPLTREAMADYLGLTLETVSRQMSALKRDGVIELEGKRHVTVP 229
FnrN R. leguminosarum  AEP-----QTATSTAFDLPLSRAEIADFLGLTIETVSRQMTRLRKIGVIRIENFRHIIVP 229
FixK B. diazoefficiens LL-AL----KGASDTVPLPMSRQDIADYLGLTIETVSRTFTKLERHGAIAII-HGGISLL 226
AadR R. palustris      LA-RL----EGVTKTVSLPMGRQDIADFLGLTIETVSRTFTKLEREKLIVIV-PDGVRVL 228
Fnr R. etli            AE-------TATSTAFDLPLSRAEIADFLGLTIETVSRQLTRLRKEGVIRIENIRHITVP 227
Btr B. pertussis       YAALG-----YSSTEFVLRMSREEIGNYLGLTLETVSRLFSRFGREGLIRIN-QREVRLI 228
AcpR Azoarcus sp.      FTARG-----FSPAEFHLRMTREEIGSYLGLKLETVSRAFSKFQDDGLIAVQ-QKHIRIL 232
Fnr A. aromaticum      FTARG-----FSAAEFHLRMTREEIGSYLGLKLETVSRAFSKFQEEGLIAVQ-QKHIRIL 232
Fnr E. coli            FAQRG-----FSPREFRLTMTRGDIGNYLGLTVETISRLLGRFQKSGMLAVK-GKYITIE 234
Anr P. aeruginosa      FRARG-----FSAQQFRLAMSRNEIGNYLGLAVETVSRVFTRFQQNGLISAE-GKEVHIL 229
                                      . * : *  :.. ***  **:** .  :     :       : :

FnrN TFA               DRAELKALAG--------- 231
FixK TFA               DPRGIGALACGIAA----- 240
FnrL R. sphaeroides    DFARLLEEAGDDSDGGLPV 248
FnrL R. capsulatus     DMDRLLIEAGDDSDGGLIA 248
FnrN R. leguminosarum  DMDELERMISA-------- 240
FixK B. diazoefficiens DPARVEALAAA-------- 237
AadR R. palustris      DPKRFDALAAA-------- 239
Fnr R. etli            DMDALAKKISG-------- 238
Btr B. pertussis       DLPGLKQLIGQESC----- 242
AcpR Azoarcus sp.      DIIGLKRLIQHPSPRP--- 248
Fnr A. aromaticum      NICGLKKLIHHPSPRG--- 248
Fnr E. coli            NNDALAQLAGHTRNVA--- 250
Anr P. aeruginosa      DSIELCALAGGQLEG---- 244
                       :   .
```

Supplementary Figure S1. Alignment of Fnr proteins of different bacteria with FnrN and FixK of TFA. The figure shows the alignment of FrnN and FixK with FnrL of *Rhodobacter sphaeroides* (RSP_0698), FnrL of *Rhodobacter capsulatus* (O33961_RHOCA), FnrN of *Rhizobium leguminosarum* (RL2818), FixK of *Bradyrhizobium diazoefficiens* (bll6061), AadR of *Rhodopseudomonas palustris* (RPA4234), Fnr of

Rhizobium etli (RHE_PD00216), Btr of *Bordetella pertussis* (BP1197), AcpR of *Azoarcus* sp. (AzCIB_3092), Fnr of *Aromatoleum aromaticum* (ebA5149), Fnr of *Escherichia coli* (b1334) and Anr of *Pseudomonas aeruginosa* (PA1544). The 4 most conserved cysteines of the ligand binding region are highlighted in blue while the conserved glutamic acid, serine and arginine of the second helix -marked with red letters- of the helix-turn-helix domain of the DNA binding region are highlighted in pink, green and red respectively. Identical amino acids are indicated by asterisks (*), high similarity amino acids are indicated by colons (:) and low similarity amino acids by dots (.). The aminoacidic sequence of the Fnr proteins belonging to different α-, β- and γ-*Proteobacteria* was obtained from Uniprot online database. The alignments of these 11 proteins with FnrN and FixK proteins of TFA was performed using Clustal Omega, using the align tool provided by Uniprot webpage.

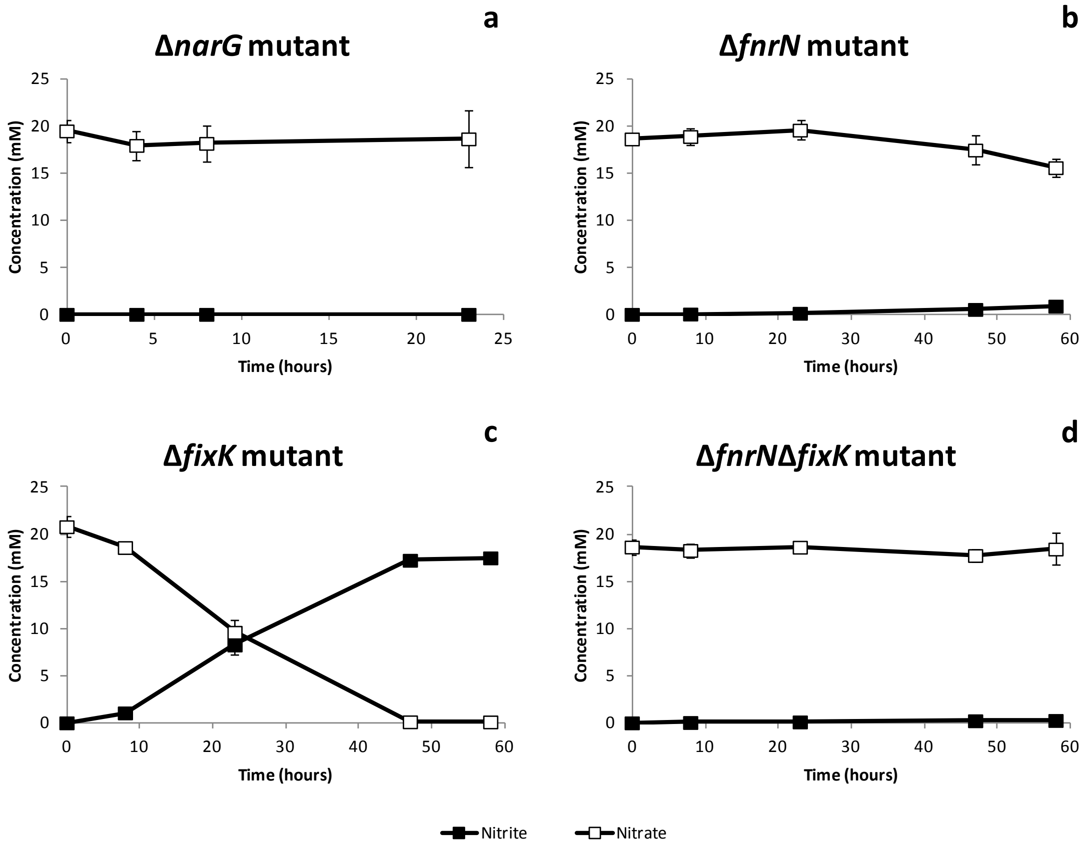

Supplementary Figure S2. Nitrate and nitrite concentrations along time during anaerobic incubation of *S. granuli* strains MPO253 (Δ*narG*), MPO250 (Δ*fnrN*), MPO251

(Δ*fixK*) and MPO252 (Δ*fnrN*Δ*fixK*). The graphics show nitrate (white squares) and nitrite (black squares) along time during anaerobic incubation in mineral medium with β-HB 40 Mm and sodium nitrate 20 mM of *S. granuli* mutant strains Δ*narG* (a), Δ*fnrN* (b), Δ*fixK* (c) and Δ*fnrN*Δ*fixK* (d). Graphics represent the mean ± SD of 3 biological replicates.

Supplementary References

1 Dorn, E., Hellwig, M., Reineke, W. & Knackmuss, H. J. Isolation and characterization of a 3-chlorobenzoate degrading pseudomonad. *Arch Microbiol* **99**, 61-70, doi:10.1007/BF00696222 (1974).

2 Gonzalez-Flores, Y. E., de Dios, R., Reyes-Ramirez, F. & Santero, E. The response of *Sphingopyxis granuli* strain TFA to the hostile anoxic condition. *Sci Rep* **9**, 6297, doi:10.1038/s41598-019-42768-9 (2019).

3 Yuste, L. *et al.* Growth phase-dependent expression of the *Pseudomonas putida* KT2440 transcriptional machinery analysed with a genome-wide DNA microarray. *Environ Microbiol* **8**, 165-177, doi:10.1111/j.1462-2920.2005.00890.x (2006).

4 Lindner, R. & Friedel, C. C. A comprehensive evaluation of alignment algorithms in the context of RNA-seq. *PLoS One* **7**, e52403, doi:10.1371/journal.pone.0052403 (2012).

5 García-Romero, I. *et al.* Genomic analysis of the nitrate-respiring *Sphingopyxis granuli* (formerly *Sphingomonas macrogoltabida*) strain TFA. *BMC Genomics* **17**, 93, doi:10.1186/s12864-016-2411-1 (2016).

6 Fischer, M., Alderson, J., van Keulen, G., White, J. & Sawers, R. G. The obligate aerobe *Streptomyces coelicolor* A3(2) synthesizes three active respiratory nitrate reductases. *Microbiology* **156**, 3166-3179, doi:10.1099/mic.0.042572-0 (2010).
